# Evaluation of log *P*, p*K*_a_, and log *D* predictions from the SAMPL7 blind challenge

**DOI:** 10.1007/s10822-021-00397-3

**Published:** 2021-06-24

**Authors:** Teresa Danielle Bergazin, Nicolas Tielker, Yingying Zhang, Junjun Mao, M. R. Gunner, Karol Francisco, Carlo Ballatore, Stefan M. Kast, David L. Mobley

**Affiliations:** 1 Department of Pharmaceutical Sciences, University of California, Irvine, Irvine, CA 92697 USA; 2 Department of Chemistry, University of California, Irvine, Irvine, CA 92697 USA; 3 Department of Physics, The Graduate Center, City University of New York, New York, 10016 USA; 4 Department of Physics, City College of New York, New York, 10031 USA; 5 Skaggs School of Pharmacy and Pharmaceutical Sciences, University of California, San Diego, Ja Jolla, CA 92093-0756 USA; 6 Physikalische Chemie III, Technische Universität Dortmund, Otto-Hahn-Str. 4a, 44227 Dortmund, Germany

**Keywords:** log *P*, SAMPL, Free energy calculations, p*K*_a_

## Abstract

The Statistical Assessment of Modeling of Proteins and Ligands (SAMPL) challenges focuses the computational modeling community on areas in need of improvement for rational drug design. The SAMPL7 physical property challenge dealt with prediction of octanol-water partition coefficients and p*K*_a_ for 22 compounds. The dataset was composed of a series of N-acylsulfonamides and related bioisosteres. 17 research groups participated in the log *P* challenge, submitting 33 blind submissions total. For the p*K*_a_ challenge, 7 different groups participated, submitting 9 blind submissions in total. Overall, the accuracy of octanol-water log *P* predictions in the SAMPL7 challenge was lower than octanol-water log *P* predictions in SAMPL6, likely due to a more diverse dataset. Compared to the SAMPL6 p*K*_a_ challenge, accuracy remains unchanged in SAMPL7. Interestingly, here, though macroscopic p*K*_a_ values were often predicted with reasonable accuracy, there was dramatically more disagreement among participants as to which microscopic transitions produced these values (with methods often disagreeing even as to the sign of the free energy change associated with certain transitions), indicating far more work needs to be done on p*K*_a_ prediction methods.

**Supplementary Information:**

The online version contains supplementary material available at 10.1007/s10822-021-00397-3.

## Introduction

Computational modeling aims to enable molecular design, property prediction, prediction of biomolecular interactions, and provide a detailed understanding of chemical and biological mechanisms. Methods for making these types of predictions can suffer from poor or unpredictable performance, thus hindering their predictive power. Without a large scale evaluation of methods, it can be difficult to know what method would yield the most accurate predictions for a system of interest. Large scale comparative evaluations of methods are rare and difficult to perform because no individual group has expertise in or access to all relevant methods. Thus, methodological studies typically focus on introducing new methods, without extensive comparisons to other methods.

The Statistical Assessment of Modeling of Proteins and Ligands (SAMPL) challenges tackle modeling areas in need of improvement, focusing the community on one accuracy-limiting problem at a time. In SAMPL challenges, participants predict a target property such as solvation free energy, given a target set of molecules. Then the corresponding experimental data remains inaccessible to the public until the challenge officially closes. By focusing on specific areas in need of improvement, SAMPL helps drive progress in computational modeling.

**Fig. 1:**
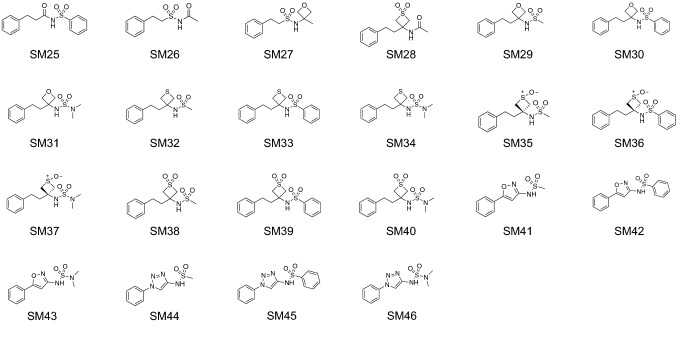
Structures of the 22 molecules used for the SAMPL7 physical property blind prediction challenge. Log of the partition coefficient between n-octanol and water was determined via potentiometric titrations using a Sirius T3 instrument. p*K*_a_ values were determined by potentiometric titrations using a Sirius T3 instrument. Log of the distribution coefficient between n-octanol and aqueous buffer at pH 7.4 were determined via potentiometric titrations using a Sirius T3 instrument, except for compounds SM27, SM28, SM30-SM34, SM36-SM39 which had log *D*_7.4_ values determined via shake-flask assay. PAMPA assay data includes effective permeability, membrane retention, and log of the apparent permeability coefficient. Permeabilities for compounds SM33, SM35, and SM39 were not determined. Compounds SM35, SM36 and SM37 are single *cis* configuration isomers. All other compounds are not chiral

Here, we report on a SAMPL7 physical property challenge that focused on octanol-water partition coefficients (log *P*) and p*K*_a_ for the series of molecules shown in Fig. 1. The p*K*_a_ of a molecule, or the negative logarithm of the acid-base dissociation constant, is related to the equilibrium constant for the dissociation of a particular acid into its conjugate base and a free proton. The p*K*_a_ also corresponds to the pH at which the corresponding acid and its conjugate base each are populated equally in solution. Given that the p*K*_a_ corresponds to a transition between specific protonation states, a given molecule may have multiple p*K*_a_ values.

The p*K*_a_ is an important physical property to take into account in drug development. The p*K*_a_ value is used to indicate the strength of an acid. A lower p*K*_a_ value indicates a stronger acid, indicating the acid more fully dissociates in water. Molecules with multiple ionizable centers have multiple p*K*_a_ values, and knowledge of the p*K*_a_ of each of the ionizable moieties allows for the percentage of ionised/neutral species to be calculated at a given pH (if activity coefficients are known/assumed). p*K*_a_ plays a particularly important role in drug development because the ionization state of molecules at physiological pH can have important ramifications in terms of drug-target interactions (e.g., ionic interactions) and/or by influencing other key determinants of drug absorption, distribution, metabolism and excretion (ADME) [1], such as lipophilicity, solubility, membrane permeability and plasma protein binding [2].

Accurate p*K*_a_ predictions play a critical role in molecular design and discovery as well since p*K*_a_ comes up in so many contexts. For example, inaccurate protonation state predictions impair the accuracy of predicted distribution coefficients such as those from free energy calculations. Similarly, binding calculations can be affected by a change in protonation state [3]. If a ligand in a protein-ligand system has a different protonation state in the binding pocket compared to when the molecule is in the aqueous phase, then this needs to be taken into account in the thermodynamic cycle when computing protein-ligand binding affinities.

**Fig. 2:**
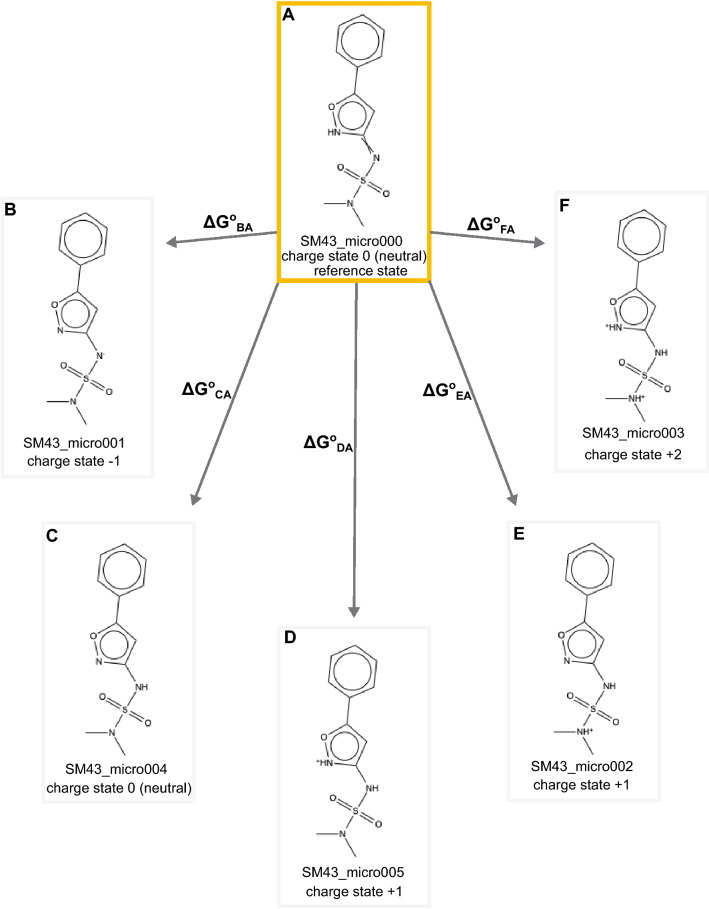
For each molecule in the SAMPL7 p*K*_a_ challenge we asked participants to predict the relative free energy between our selected neutral reference microstate and the rest of the enumerated microstates for that molecule. In this case, we asked for the relative state free energy including the proton free energy, which could also be called the reaction free energy for the microstate transition which has the reference state as the reactant and the alternate state as the product. Using SM43 as an example, participants were asked to predict the relative free energy between SM43_micro000 (our selected neutral microstate highlighted in yellow) and all of the other enumerated microstates (SM43_micro001–SM43_micro005) for a total of 5 relative state free energies ($\Delta$G_BA_, $\Delta$G_CA_, $\Delta$G_DA_, $\Delta$G_EA_, $\Delta$G_FA_). Some transitions involved a change in a single protonation state (e.g. the D–A transition of Figure 2) or tautomer (e.g. the C–A transition of Figure 2). A few cases involved a change of multiple protons (e.g. the F–A transition of Figure 2). All transitions were defined as *away* from the neutral reference state. Distinct microstates are defined as all tautomers of each charge state. For each relative free energy prediction reported, participants also submitted the formal charge after transitioning from the selected neutral state to the other state. For example, the reported charge state after transitioning from SM43_micro000 to SM43_micro001 would be − 1, SM43_micro000 to SM43_micro004 would be 0 (these are tautomers of each other), SM43_micro000 to SM43_micro005 would be + 1, and SM43_micro000 to SM43_micro003 would be + 2

Multiprotic molecules, and those with multiple tautomeric states, have two types of p*K*_a_, microscopic and macroscopic. The *microscopic* p*K*_a_ applies to a specific transition or equilibrium between microstates, i.e. for a transition between a specific tautomer at one formal charge and that at another formal charge (e.g. two states at different formal charges in Figure 2). It relates to the acid dissociation constant associated with that specific transition. As a special case, a microscopic p*K*_a_ sometimes refers to the p*K*_a_ of deprotonation of a single titratable group while all the other titratable and tautomerizable functional groups of the same molecule are held fixed, but this might possibly not reflect the dominant deprotonation pathway of a given acidic tautomer if the base state possesses energetically favored alternate tautomers. There is no p*K*_a_ between two tautomers with the same formal charge because they have the same number of protons so their relative probability is independent of pH. The pH-independent free energy difference between them determines their relative population [4].

At some level, the macroscopic p*K*_a_ can be thought of as describing the acid dissociation constant related to the loss of a proton from a molecule regardless of which functional group the proton is dissociating from, but it may be more helpful to think of it (in the case of polyprotic molecules) as a macroscopic observable describing the collective behavior of various tautomeric states as the dominant formal charge of the molecule shifts. In cases where a molecule has only a single location for a titratable proton, the microscopic p*K*_a_ becomes equal to the macroscopic p*K*_a_.

In the current challenge, we explored how well methods could predict macroscopic p*K*_a_’s through microscopic p*K*_a_ calculations.

The partition coefficient (log *P*) and the distribution coefficient (log *D*) are relevant to drug discovery, as they are used to describe lipophilicity. Lipophilicity influences drug-target and off-target interactions through hydrophobic interactions, and relatively high lipophilicity results in reduced aqueous solubility and increased likelihood of metabolic instability [5].

Prediction of partitioning and distribution has some relevance to drug distribution. Particularly, partitioning and distribution experiments involve a biphasic system with separated aqueous and organic phases, such as water and octanol, so such experiments have some of the features of the interface between blood or cytoplasm and the cell membrane [6, 7] and thus improved predictive power for partitioning and distribution may pay off with an improved understanding of such in vivo events.

Methods to predict log *P*/log *D* may also use (and test) some of the same techniques which can be applied to binding predictions. Both types of calculations can use solvation free energies and partitioning between environments (though this could be avoided by computing the transfer free energy). Such solute partitioning models are simple test systems for the transfer free energy of a molecule to a hydrophobic environment of a protein binding pocket, without having to account for additional specific interactions which are present in biomolecular binding sites. Thus partitioning and distribution calculations allow separating force-field accuracy from errors related to conformational sampling of proteins and protonation state predictions of proteins and ligands.

The log *P* is usually defined as the equilibrium concentration ratio of the neutral state of a substance between two phases:

1 $$\begin{aligned} \log P = \log _{10} K_\text {ow} = \log _{10} \frac{[\text {unionized~solute}]_\text {octanol}}{[\text {unionized~solute}]_\text {water}} \end{aligned}$$

Strictly speaking, this definition of the partition coefficient *P* as a thermodynamic equilibrium constant is independent of total solute concentration in the infinite dilution limit only. This reference state is commonly assumed in physics-based prediction models. The log *P* prediction challenge explores how well current methods are able to model the transfer free energy of molecules between different solvent environments without any complications coming from predicting protonation states.

### Motivation for the log *P* and p*K*_a_ challenge

Previous SAMPL challenges have looked at the prediction of solvation free energies [8–12], guest-host [13–19] and protein-ligand binding affinities [20–26], p*K*_a_ [27–33], distribution coefficients [34–37], and partition coefficients [38–41]. These challenges have helped uncover sources of error, pinpoint the reasons various methods performed poorly or well and their strengths and weaknesses, and facilitate dissemination of lessons learned after each challenge ends, ultimately leading to improved methods and algorithms.

Several past challenges focused on solvation modeling in order to help address this accuracy-limiting component of protein-ligand modeling. The SAMPL0 through SAMPL4 challenges included hydration free energy prediction, followed by cyclohexane-water distribution coefficient prediction in SAMPL5, and octanol-water distribution coefficient prediction in SAMPL6. Large errors were observed in the SAMPL5 cyclohexane-water log *D* prediction challenge due to tautomers and protonation states not being taken into account [29, 42] or adequately handled. Many participants reported log *P* predictions in place of log *D* predictions, in part because the different ionization states of the molecules were thought not to be particularly relevant in the challenge, but this proved not to be the case. Methods that treated multiple protonation and tautomeric states and incorporated p*K*_a_ corrections (which relies on accurate p*K*_a_ prediction) in their predictions performed better [42].

In order to pinpoint sources of error in log *D* predictions, separate log *P* and p*K*_a_ challenges were organized for SAMPL6 [27, 38, 43, 44]. Better prediction performance was seen in the SAMPL6 octanol-water log *P* challenge compared to the SAMPL5 cyclohexane-water log *D* challenge. Performance improved in SAMPL6 for several reasons. First, the latter challenge avoided the p*K*_a_ prediction problem. Second, far more experimental training data was available (aiding empirical and implicit QM methods). Finally, the more narrow chemical diversity in SAMPL6 may have helped participants. For the present SAMPL7 physical properties challenge, we focused on assessing the accuracy of log *P* and p*K*_a_ predictions, and then combined p*K*_a_ and log *P* predictions to obtain log *D* predictions.

### Historical SAMPL p*K*_a_ performance

During the SAMPL6 challenge a broad range of conceptually different empirical and physics–based computational methods were used to predict p*K*_a_ values, as discussed in the overview paper [43]. To provide some context for the results of the SAMPL7 challenge the main results are summarized here.

The empirical approaches used during SAMPL6 can be divided into three categories, Database Lookup (DL), Linear Free Energy Relationship (LFER), and Quantitative Structure–Property/Machine Learning (QSPR/ML) approaches [12]. The physical approaches can be divided into pure quantum–mechanical (QM) methods, QM with a linear empirical correction (QM+LEC) to account for the free energy of the proton in solution or potential systematic errors caused by the chosen method, and QM in combination with molecular mechanics (QM+MM). Generally speaking, the empirical methods require significantly less computational effort than their physics–based counterparts once they are parameterized.

The best–performing models included four empirical methods and one QM-based model. These five methods were able to predict the acidity constants of the challenge compounds to within 1 p*K*_a_ unit. In fact, while most empirical models—except for the DL and two of the five QSPR/ML approaches—were able to predict the acidity constants to within about 1.5 p*K*_a_ units, the range of predictions was much wider for the QM-based models.

In SAMPL6, many groups submitted multiple predictions to test the performance of different variations using the same basic methodology, such as exploring different levels of theory, model parameters, or conformational ensembles.

Well–performing empirical models included both LFER methods, such as ACD/pKa Classic (submission ID *xmyhm*) and Epik Scan (*nb007*), and QSPR/ML methods such as MoKa (*nb017*) and S+pKa (*gyuhx*), all performing with root mean square errors (RMSE) between 0.73 and 0.95 p*K*_a_ units [45–48]. These well-established tools thus demonstrated their reliability and quality.

Among the physics–based models, the most straightforward approach involved calculation of the acidity constants without any empirical corrections, including the experimental value for the free energy of solvation of the proton [49]. One group applied different calculation schemes to the compounds of the SAMPL6 challenge that differed in the use of gas phase and/or solution phase geometries as well as additional high–level single point gas phase calculations [30]. While the results achieved by this method were quite promising, with an initial RMSE of 1.77 p*K*_a_ units (*ryzue*) that could be improved to 1.40 by including a standard state correction and a different value for the free energy of the proton, the authors also showed the effectiveness of a simple linear regression scheme to correct the raw acidity constants. In this case the RMSE of the best-performing model decreased further from 1.40 to 0.73 p*K*_a_ units after regression.

This type of empirical correction was used by most QM-based approaches, including the best–performing method of the SAMPL6 challenge [43], improving some systematic deficiencies of the QM level of theory and basis sets and accounting for the proton’s solvation free energy. The best-performing QM+LEC method, *xvxzd*, achieved an RMSE of 0.68 p*K*_a_ units during the challenge using the COSMO-RS solvation model. This also made it the best–performing model overall, with two other methods using the same solvation model only slightly worse (*yqkga* and *8xt50*, with RMSEs of 1.01 and 1.07 p*K*_a_ units, respectively [32, 43, 50]).

A QM+LEC method using a different solvation approach, EC-RISM, only achieved an RMSE of 1.70 p*K*_a_ units for the submitted model (*nb001*), but a post-submission optimization of the conformer generation workflow and the electrostatic interactions improved the RMSE to 1.13, which is more in line with the other well–performing QM+LEC methods [31]. The CPCM implicit solvation model was used by one group  [28, 43] and performed only slightly worse than COSMO-RS (RMSEs from the paper do not agree with official numbers. Only officially submitted ones are discussed here). For these two models, differing only by training either a single LEC for all compounds (*35bdm*) or two separate LECs for deprotonations of neutral compounds to anions and deprotonations of cations to neutral compounds (*p0jba*), the RMSEs were 1.72 and 1.31 p*K*_a_ units, respectively. These results show that accurate p*K*_a_ values can be predicted when using the QM+LEC approach with different solvation models.

A slightly different approach was used by one participant (*0wfzo*) where QM calculations of the free energy of deprotonation and thermodynamic integration, an MM method, were combined to calculate the difference of the solvation free energies between the acid and its conjugate base [33]. This approach yielded an average level of performance, with an RMSE of 2.89 for the macroscopic acidity constants calculated from the submitted microscopic acidity constants, excluding two compounds (SM14 and SM18) from the analysis as they exhibited multiple p*K*_a_ values too close to each other.

### Approaches to predicting small molecule p*K*_a_’s

Calculations of aqueous p*K*_a_ values have a long history in computational chemistry, with methods ranging from direct quantum-mechanical approaches for determining the free energy of protonated and deprotonated species in solution using explicit, implicit, or hybrid solvation models, to continuum electrostatics-based computations of relative p*K*_a_ shifts, and empirical or rule-based algorithms, as summarized in a number of review articles, e.g. Alongi et al. [51],and Liao et al. [52] and in the SAMPL6 overview papers [27, 43].

Computational methods typically designate tautomeric states (“microstates”) for acid and base forms of a compound separated by a unit charge upon (de-)protonation. Their free energies can be linked individually in a pair-wise manner (“microstate transitions”) to yield so–called microstate p*K*_a_ values from which the macroscopic p*K*_a_ can be determined [53]. Alternatively, the tautomer free energies, combined across the underlying conformational states, contribute to the ratio of partition functions representing acid and base forms, allowing the direct calculation of macroscopic acidity constants [54]. A complication arises if, as is common practice with quantum-mechanical approaches, the difference of solution-state (standard) free energies for differently charged species, $G(\text {A}_{\text {aq}}^{-})$ and $G(\text {HA}_{\text {aq}})$ for a general reaction

2 $$\begin{aligned} \text {HA}\rightarrow \text {A}^{-}+\text {H}^{+} \end{aligned}$$

are scaled by a “slope” factor *m* and augmented by an intercept parameter *b* to account for the free energy of the proton, yielding a regression equation, given here for microstate *j* of the base and *k* of the acid form, respectively,

3 $$\begin{aligned} \text {p}K_{a,\,jk}=b+\frac{m}{RT\ln 10}[G_{j}(\text {A}^{-})-G_{k}(\text {HA})] \end{aligned}$$

where slope and intercept are typically adjusted with respect to databases of experimental p*K*$_{\text {a}}$ values [54] and *RT* has the usual thermodynamic meaning. Here *G* denotes the Gibbs free energy, but a similar expression would hold for Helmholtz free energy depending on the choice of ensemble.

As derived in Tielker et al. [54], statistics over all connected microstates (in the “state transition” (ST) approach) and *a priori* partition function summation (in the “partition function” (PF) approach) are identical if and only if $m=1$, though in practice the difference is usually negligible.

For the SAMPL7 p*K*_a_ challenge, participants were required to submit predictions in a novel format, reporting transition free energies between microstates as in the “$\Delta G^{0}$” formalism outlined in Gunner et al. [55] (and similar to the work of Selwa et al. [28]). Here, the pH–dependent free energy change between “states” *k* and *j* is defined by rewriting the well-known Henderson-Hasselbalch equation for, e.g., the general reaction (Eq. 3) in the form

4 $$\begin{aligned} \Delta G_{jk}\left( \text {pH}\right) =\Delta m_{jk}C_{\text {units}}\left( \text {pH}-\text {p}K_{a,\,jk}\right) \end{aligned}$$

with $C_{\text {units}}=RT\ln 10$ and, for a transition away from the reference state which involves loss of a proton, $\Delta m_{jk}=-1$, denoting the charge difference between the “reference state” *k* (second index, usually taken as a selected neutral microstate, in this case $\text {HA}_{\text {aq}}$) and the target state *j*.

For the thermodynamic standard state at $\text {pH}=0$ we can write

5 $$\begin{aligned} \Delta G_{jk}^{0}=-\Delta m_{jk}C_{\text {units}}\text {p}K_{a,\,jk} \end{aligned}$$

which shows that $\Delta G_{jk}^{0}$ can be identified with a formal free energy of reaction. An advantage of this approach is that closed thermodynamic cycles by summing over $\Delta G_{jk}^{0}$ with identical reference *k* would add to zero for consistent computational methods, which can serve as an added value for testing theoretical frameworks [55].

The macroscopic p*K*_a_ is obtained by computing the total fraction of all microstates with charge *q* and $j\in q$ via

6 $$\begin{aligned} x_{j\in q}(\text {pH})=\frac{\exp [-\Delta G_{j\in q,k}(\text {pH})/RT]}{\sum _{i}\exp [-\Delta G_{ik}(\text {pH})/RT]} \end{aligned}$$

and solving, usually numerically, for the pH at which

7 $$\begin{aligned} x_{j\in q\left( 1\right) }\left( \text {pH}\right) =x_{j\in q\left( 2\right) }\left( \text {pH}\right) \end{aligned}$$

for adjacent net charges *q*(1) and *q*(2). At this pH, $\text {p}K_{\text {a}}=\text {pH}$ for these particular charge states, and this approach constitutes a formal “titration”.

Outlining the connection between the $\Delta G^{0}$ and the ST and PF formalisms [54] is useful for practitioners who directly compute microstate free energies (including corresponding tautomerization free energies for which no p*K*_a_ is defined) or microstate transition p*K*_a_ values for single deprotonation reactions where a specific reaction direction is by definition implied. The general algorithm is as follows, with subscript order $\text {p}K_{a,\,jk}$ implying the reaction $j\rightarrow k^{-}+\text {H}^{+}$ for any total charge on *j* and subscript order $\Delta G_{jk}^{0}$ meaning the reaction $k(+m\text {H}^{+})\rightarrow j(+n\text {H}^{+})$ with neutral *k*. For all states *i* not equal to the neutral reference microstate *k* we have

- If $$q(i)=0$$, $$\Delta G_{ik}^{0}=m\Delta G^{0}(k\rightarrow i)$$
- If $q(i)-q(k)=+1$ (the reaction is $k+\text {H}^{+}\rightarrow i^{+})$, then $\Delta G_{ik}^{0}=-C_{\text {units}}\text {p}K_{a,ik}$
- If $q(i)-q(k)=-1$ (the reaction is $k\rightarrow i^{-}+\text {H}^{+})$, then $\Delta G_{ik}^{0}=+C_{\text {units}}\text {p}K_{a,ki}$
- If $q(i)-q(k)=+2$ (the reaction is $k+2\text {H}^{+}\rightarrow i^{2+}$ via the individual reactions $k+\text {H}^{+}\rightarrow j^{+}$ and $j^{+}+\text {H}^{+}\rightarrow i^{2+})$, then $\Delta G_{ik}^{0}=-C_{\text {units}}(\text {p}K_{a,jk}+\text {p}K_{a,ij})$
- If $q(i)-q(k)=-2$ (the reaction is $k\rightarrow i^{2-}+2\text {H}^{+}$ via the individual reactions $k\rightarrow j^{-}+\text {H}^{+}$ and $j^{-}\rightarrow i^{2-}+\text {H}^{+})$, then $\Delta G_{ik}^{0}=+C_{\text {units}}(\text {p}K_{a,kj}+\text {p}K_{a,ji})$

This scheme is readily generalized to changes of more than two unit charges. The scaling by the factor *m* in (a) guarantees consistency over closed thermodynamic cycles in the common case of non-zero slope parameter for QM-based models.

To demonstrate how macroscopic p*K*_a_ values computed this way relate to ST and PF results it is instructive to treat the simple example of a two-tautomer acid in equilibrium with a single-tautomer base, i.e.

8 $$\begin{aligned} \text {HA}_{1}\overset{K_{\text {a},1}}{\rightarrow }\text {A}^{-}+\text {H}^{+}, \text {HA}_{2}\overset{K_{\text {a},2}}{\rightarrow }\text {A}^{-}+\text {H}^{+} \end{aligned}$$

for which Eq. (3) yields  [54]

9 $$\begin{aligned} K_{\text {a}}^{\text {ST}}=\left( \frac{1}{K_{\text {a},1}}+\frac{1}{K_{\text {a},2}}\right) ^{-1}=10^{-b}\frac{\exp \left[ -mG\left( \text {A}^{-}\right) /RT\right] }{\exp \left[ -mG\left( \text {HA}_{1}\right) /RT\right] +\exp \left[ -mG\left( \text {HA}_{2}\right) /RT\right] } \end{aligned}$$

Following the algorithm for $\Delta G_{jk}^{0}$ above with $\text {HA}_{1}$ assumed as neutral reference and augmenting the pH dependence according to Eq. (4) we have

10 $$\begin{aligned}&\Delta G\left( \text {HA}_{1}\right) =0 \end{aligned}$$

11 $$\begin{aligned}&\Delta G\left( \text {HA}_{2}\right) =m\left[ G\left( \text {HA}_{2}\right) -G\left( \text {HA}_{1}\right) \right] \end{aligned}$$

12 $$\begin{aligned} \Delta G\left( \text {A}^{-}\right) =-C_{\text {units}}\left( \text {pH}-\text {p}K_{\text {a},1}\right) =m\left[ G\left( \text {A}^{-}\right) -G\left( \text {HA}_{1}\right) \right] -C_{\text {units}}\left( \text {pH}-b\right) \end{aligned}$$

From Eq. 5 and equating neutral and charged molar fractions it follows from $x(\text {HA})=x(\text {A}^{-})$

13 $$\begin{aligned} 1+\exp \left\{ -m\left[ G\left( \text {HA}_{2}\right) -G\left( \text {HA}_{1}\right) \right] /RT\right\} =10^{-b}\exp \left\{ +m\left[ G\left( \text {HA}_{1}\right) -G\left( \text {A}^{-}\right) \right] /RT\right\} /K_{\text {a}} \end{aligned}$$

which, upon rearrangement and comparison with (9), yields

14 $$\begin{aligned} K_{\text {a}}=K_{\text {a}}^{\text {ST}} \end{aligned}$$

Generalization to more complex tautomeric mixtures and arbitrary reference states is possible, the latter by recognizing that these would only imply cancelling additive constants. The $\Delta G^{0}$ and ST formalisms are therefore equivalent, as is the PF approach for $m=1$.

### Approaches to predicting log *P*

Approaches for predicting octanol-water log *P* values include physical modeling methods, such as quantum mechanics (QM) and molecular mechanics (MM) approaches, and empirical knowledge-based prediction methods, such as contribution-type approaches. We give some brief background on these prediction methods.

QM approaches use a numerical solution of the Schrödinger equation to estimate solvation free energies and partitioning. These approaches are not practical for larger systems, so certain approximations need to be made so that they can be used for calculating transfer free energies. Methods typically represent the solvent using an implicit solvent model and make the assumption that the solute has a single or a small number of dominant conformations in the aqueous and non-aqueous phase. The accuracy of predictions can be influenced by the basis set, level of theory, and the tautomer used as input. Implicit solvent models are used to represent both octanol and water, and these models are often highly parameterized on experimental solvation free energy data. The abundance of training data contributes to the success of QM methods, much like empirical prediction methods. Solvent models such as SMD [56], the SM-n series of models [57], and COSMO-RS [37, 58–61] are frequently used by SAMPL participants.

MM approaches use a force field which gives the energy of a system as a function of the atomic positions and are usually used by SAMPL participants to compute solvation free energies and log *P* values. Force fields can be fixed charge and additive, or polarizable [36, 62], and typically include all atoms, though this need not always be the case. These approaches are usually applied by integrating the equations of motion to solve for the time evolution of the system. Force fields such as GAFF [63], GAFF2 [64], CGenFF [65], and OPLS-AA [66], and water models such as TIP3P [67], TIP4P [67], OPC3 [68] are frequently used in SAMPL challenges [38]. Free energy calculations can be combined with MM methods to give a partitioning estimate. These types of calculations often use alchemical free energy methods to estimate phase transfer via a non-physical thermodynamic cycle. Some examples of alchemical approaches include non-equilibrium switching [69, 70] and equilibrium alchemical free energy calculations [71] analyzed via thermodynamic integration [72] or BAR/MBAR estimation [73, 74], Such simulations can also use techniques like Hamiltonian replica exchange molecular dynamics.

Some limitations of MM approaches include the accuracy of the force field and the limitation that motions can only be captured in simulations that are faster than simulation timescales. The state of the molecule that is used as input is also important—usually, a single tautomer/protonation state is selected and held fixed throughout the simulation, which can introduce errors if the wrong state was selected or if there are multiple relevant states.

Empirical prediction models are trained on experimental data and can be used to quickly characterize large virtual libraries. These include additive group methods, such as fragment- or atom-contribution approaches, and quantitative structure-prop erty relationship (QSPR) methods. In atom contribution approaches, the log *P* is equal to the sum of contributions from the individual atom types multiplied by the number of occurrences of each in the molecule. These methods make the assumption that each atom contributes a certain amount to the solvation free energy and that these contributions are additive to the log *P* . In fragment (or group) contribution approaches, the log *P* is equivalent to the sum of the contributions from the fragment groups (more than a single atom), and typically uses correction terms that consider intramolecular interactions. These approaches are generally calculated by adding together the sum of the fragment contributions times the number of occurrences and the sum of the correction contributions times the number of occurrences in the molecule. The other class of empirical log *P* prediction approaches relies on QSPR. In QSPR, molecular descriptors are calculated and then used to make log *P* predictions. Descriptors can vary in complexity—some rely on simple counts of heteroatoms and carbon, while others are derived from correlating the 3D shape, electrostatic, and hydrogen bonding characteristics with the log *P* of the molecule. To find the log *P*, a regression model gets derived by fitting the descriptor contributions to experimental data. Machine learning approaches such as random forest models, deep neural network models, Gaussian processes, support vector machines, and ridge regression [75, 76] belong under this category.

Empirical methods tend to benefit from a large and diverse training set, especially when there’s a large body of experimental data to train on, such as octanol-water data like in the present and previous log *P* challenge [38]. However, empirical methods can experience problems if a training set has an underrepresented functional group. Additionally, these techniques are geared towards partitioning predictions, and, unlike physical-based methods, are not able to be applied to protein-ligand binding.

## Challenge design and evaluation

### General challenge structure

The SAMPL7 physical property challenge focused on p*K*_a_, partitioning, and permeability. As reported separately, KF and CB collected a set of measured water-octanol log *P*, log *D*, and p*K*_a_ values for 22 compounds, along with PAMPA permeability values [77]. Since this was our first time hosting a permeability challenge, and these calculations remain challenging for many methods, we did not have enough participants to form meaningful conclusions (one participant submitted two sets of predictions in total) so the challenge is not discussed in this paper, but we provide a link to the challenge’s GitHub page (https://github.com/samplchallenges/SAMPL7/tree/master/physical_property/permeability).

The SAMPL7 challenge molecules had weights that ranged from 227 to 365 Da, and varied in flexibility (the number of non-terminal rotatable bonds ranged from 3 to 6). The dataset had experimental log *P* values in the range of 0.58–2.96, p*K*_a_ values in the range of 4.49–11.93, and log *D* values in the range of − 0.87 to 2.96. Information on experimental data collection is presented elsewhere [77].

The physical properties challenge was announced on June 29th, 2020 and the molecules and experimental details were made available at this time. Additional input files, instructions, and submission templates were made available afterward and participant submissions were accepted until October 8th, 2020. Following the conclusion of the blind challenge, the experimental data was made public on October 9th, 2020, and results were discussed in a virtual workshop (on November 2–5, 2020) (SAMPL Community Zenodo page https://zenodo.org/communities/sampl/?page=1&size=20).

A machine-readable submission file format was specified for blind submissions. The submission files included fields for naming the method of the computational protocol, listing the average compute time across all of the molecules, detailing the computing and hardware used, listing the major software packages and the versions that were used, and a free text method section for providing the detailed documentation of each method, the values of key parameters with units, and to explain how statistical uncertainties were estimated. There was also a field where participants indicated whether or not they wanted their submission formally evaluated. In addition to their predictions, participants were asked to estimate the statistical error [expressed as a standard error of the mean (SEM)] associated with their predictions, and the uncertainty of their model. The SEM captures the statistical uncertainty of a method’s predictions, and the model uncertainty corresponds to the method’s expected prediction accuracy, which estimates how well a participant expects their predicted values will agree with experiment. Historically, model uncertainty estimates have received relatively little attention from participants, but we retain hope that participants may eventually predict useful model uncertainties since users benefit from knowing the accuracy of a predicted value.

Participants had the option of submitting predictions from multiple methods, and were asked to fill out separate template files for each different method. Each participant or organization could submit predictions from multiple methods, but could only have one ranked submission. Allowing multiple submissions gave participants the opportunity to submit prediction sets to compare multiple methods or to investigate the effect of varying parameters of a single method. All of the submissions were assigned a short descriptive method name based on the name they provided for their protocol in their submission file. This descriptive method name was used in the analysis and throughout this paper and is presented in Tables 1, 3, and 5.

**Table 1:** Method names, category, and submission type for all the log *P* calculation submissions

Method name	Category	Submission type
*ClassicalGSG DB2* [84–86]	Empirical	Blind
*TFE MLR* [87]	Empirical	Blind
*ClassicalGSG DB4* [84–86]	Empirical	Blind
*Chemprop* [88]	Empirical	Blind
*TFE-SM8-vacuum-opt*	Physical (QM)	Blind
*GROVER*	Empirical	Blind
*ClassicalGSG DB1* [84–86]	Empirical	Blind
*ffsampled_deeplearning_cl1*	Empirical	Blind
*ClassicalGSG DB3* [84–86]	Empirical	Blind
*COSMO-RS* [89]	Physical (QM)	Blind
*TFE_Attentive_FP*	Empirical	Blind
*ffsampled_deeplearning_cl2*	Empirical	Blind
*TFE-SM12-vacuum-opt*	Physical (QM)	Blind
*TFE-SM8-solvent-opt*	Physical (QM)	Blind
*REF1 ChemAxon* [80]	Empirical	Reference
*TFE IEFPCM MST* [90]	Physical (QM)	Blind
*TFE MD neat oct (GAFF/TIP4P)*	Physical (MM)	Blind
*NULL0 mean clogP FDA* [38]	Empirical	Reference
*NES-1 (GAFF2/OPC3) G*	Physical (MM)	Blind
*NES-1 (GAFF2/OPC3) J*	Physical (MM)	Blind
*NES-1 (GAFF2/OPC3) B*	Physical (MM)	Blind
*MD (GAFF/TIP3P)* [91]	Physical (MM)	Blind
*TFE wet oct (GAFF/TIP4P)*	Physical (MM)	Blind
*MD (CGenFF/TIP3P)* [91]	Physical (MM)	Blind
*EC_RISM_wet* [92]	Physical (QM)	Blind
*TFE-SMD-vacuum-opt*	Physical (QM)	Blind
*MD-EE-MCC (GAFF-TIP4P-Ew)* [93]	Physical (MM)	Blind
*TFE b3lypd3* [94]	Physical (QM)	Blind
*MD (OPLS-AA/TIP4P)* [91]	Physical (MM)	Blind
*MD LigParGen (OPLS-AA/TIP4P)* [91]	Physical (MM)	Blind
*TFE-SMD-solvent-opt*	Physical (QM)	Blind
*TFE-NHLBI-TZVP-QM*	Physical (QM)	Blind
*Ensemble EPI physprop*	Empirical	Blind
*Ensemble Martel*	Empirical	Blind
*QSPR_Mordred2D_TPOT_AutoML*	Empirical	Blind
*TFE-NHLBI-NN-IN*	Empirical	Blind

The “submission type” column indicates if submission was a blind submission (denoted by “Blind”) or a post-deadline reference or null calculation (denoted by “Reference”). The table is ordered from lowest to highest RMSE, although many consecutively listed methods are statistically indistinguishable. All calculated error statistics are available in Table S1

### log *P* challenge structure

The SAMPL7 log *P* challenge consisted of predicting the water-octanol partition coefficients of 22 molecules. Our goal was to evaluate how well current models can capture the transfer free energy of small molecules between different solvent environments through blind predictions. challenge participants were asked to predict the difference in free energy for the neutral form of each molecule between water and octanol. For the log *P* challenge, participants were required to report, for each molecule, the SAMPL7 molecule ID tag (the challenge provided neutral microstate), the microstate ID or IDs that were considered, and the predicted transfer free energy, transfer free energy SEM, and model uncertainty.

Participants were asked to categorize their methods as one of the five method categories—physical (QM), physical (MM), empirical, or mixed. Participants were asked to indicate their method based on the following definitions: Empirical models are prediction methods that are trained on experimental data, such as QSPR, machine learning models, artificial neural networks, etc. Physical models are prediction methods that rely on the physical principles of the system such as MM or QM based physical methods to predict molecular properties. Participants were asked to indicate whether their physical method was QM or MM based. Methods taking advantage of both kinds of approaches were asked to be reported as “Mixed”. If a participant chose the “Mixed” category, they were asked to explain their decision in the method description section in their submission file.

We highlighted that octanol may be found in the aqueous phase, in case participants wanted to consider this in their predictions. The mole fraction of water in octanol was measured as 0.271 ± 0.003 at 25 °C [7].

### p*K*_a_ challenge structure

The SAMPL7 p*K*_a_ challenge consisted of predicting relative free energies between microstates (microscopic p*K*_a_’s) to determine the macroscopic p*K*_a_ of 22 molecules. Our goal for the SAMPL7 p*K*_a_ challenge was to assess how well current p*K*_a_ prediction methods perform for the 22 challenge molecules through blind predictions.

We chose to have participants report relative free energies of microstates for simplicity of analysis. Particularly, for each molecule, participants were asked to predict the relative free energy, including the proton free energy, between our selected neutral reference microstate and the rest of the enumerated microstates for that molecule at a reference pH of 0 (see "Approaches to predicting small molecule p*K*_a_’s" sect. on approaches to calculating p*K*_a_). This can also be thought of as a reaction free energy for the microstate transition where the reference state is the reactant and the other microstate the product (though a proton may also be a product, depending on the direction of the transition). As an example for one molecule, we asked for the reaction free energy (relative free energy) associated with each of the reactions as seen in Figure 2. This approach differs from that used in past p*K*_a_ challenges, which typically focused on macroscopic p*K*_a_ predictions. The shift, here, helps resolve several key problems:

1. A macroscopic p*K*_a_ can be reported for the wrong microstates, leading to predictions that are accidentally correct, but fundamentally wrong because the titration referred to a different states of the molecule.
2. Analysis of p*K*_a_ predictions requires pairing calculated macroscopic p*K*_a_ values with corresponding experimental macroscopic p*K*_a_ values [43] and such pairing can be very complex without information on which states are being predicted; while pairing is still required when specific transitions are predicted, it is aided by knowing *which* transitions are predicted (e.g. a − 1 to 0 prediction from one participant can no longer accidentally be compared with a 0 to + 1 transition from another participant)
3. Ultimately, populations and free energy differences between states drive the experimental measurements, so analysis ought to focus on state populations

In this work, all possible tautomers of each ionization (charge) state are defined as distinct protonation microstates. For the p*K*_a_ challenge, participants were required to report, for each molecule and each microstate they considered, the microstate ID of the reference state (selected by challenge organizers), the microstate ID of the microstate they were considering a transition to, the formal charge for the target microstate, and the predicted free energy change associated with a transition to the target microstate (Figure 2), the relative free energy SEM, and the relative free energy model uncertainty. In many cases, the transitions to be considered were a particular physical reaction involving a change in a single protonation state or tautomer. However, in some cases transitions involved a change of multiple protons (e.g. the F–A transition of Figure 2) and thus did not involve a single protonation or deprotonation event. Additionally, all transitions were defined as *away* from the reference state (and thus some involve gaining a proton, the opposite of a typical acid dissociation event), a point which caused confusion for a number of participants.

All predictions were required to use free energy units, in kcal/mol, which was another point which caused confusion for participants, as we received predictions in several different sets of units and had to handle unit conversion after the challenge close.

Participants were asked to define and categorize their methods based on the following six method categories- experimental database lookup (DL), linear free energy relationship (LFER) [12], quantitative structure-property relationship or machine learning (QSPR/ML) [12], quantum mechanics without empirical correction (QM) models, quantum mechanics with linear empirical correction (QM+LEC), and combined quantum mechanics and molecular mechanics (QM+MM), or “Other”. If the “Other” category was chosen, participants were asked to explain their decision in the beginning of the method description section in their submission file.

#### Microstate enumeration

The SAMPL7 p*K*_a_ challenge participants were asked to predict relative free energies between microstates to determine the p*K*_a_ of molecules. We define distinct protonation microstates as all possible tautomers of each ionization (charge) state. Participants could consider any of these microstates in their predictions, and had the option of submitting others. Participants were provided a reference microstate for each compound, and asked to predict transition free energies to all microstates they viewed as relevant, relative to this reference state.

Here, we provided some enumeration of potential microstates that participants might want to consider. To do so, we used more than one toolkit to try and ensure all reasonable tautomers and protomers were included. Our microstates were generated using RDKit [78] and OpenEye QUACPAC [79] for protonation state/tautomer enumeration, and then cross checked with ChemAxon Chemicalize [80] and Schrodinger Epik [46, 81] to ensure we had not missed states. We also allowed participants to submit additional microstates they might view as important, and received one set of such submissions, which resulted in us adding a microstate with a + 1 formal charge to molecules SM31 (SM31_micro002) and SM34 (SM34_micro002). It is unclear why this state was not identified by the tools we used to enumerate microstates.

We provided participants CSV (.csv) tables which included microstate IDs and their corresponding canonical isomeric SMILES string, as well as individual MOL2 (.mol2) and SDF (.sdf) files for each individual microstate. These are available in the SAMPL7 GitHub repository.

### Combining log *P* and p*K*_a_ predictions to estimate log *D*

In the SAMPL7 challenge, log *P* and p*K*_a_ predictions were combined in order to estimate log *D*. The relationship between partition and distribution coefficients at a given pH can be computed via [82, 83]

15 $$\begin{aligned} \log D_{\text {pH}}=\log P-\log \left( 1+10^{\text {p}K\text {a}-\text {pH}}\right) \end{aligned}$$

for bases (if no deprotonation site is present or if ${\text{p}}{} \textit{K}_{\text {b}} {<} \text{p}{} \textit{K}_{\text {a}}$) and

16 $$\begin{aligned} \log D_{\text {pH}}=\log P-\log \left( 1+10^{\text {pH}-\text {p}K\text {a}}\right) \end{aligned}$$

for acidic compounds. The log *D* was calculated under the assumption that the ionic species cannot partition into the organic phase [34], which may be important in some cases (e.g. in compounds with high lipophilicity or in cases where pH is so extreme that partitioning of a charged species might become important).

### Evaluation approach

We considered a variety of error metrics when analyzing predictions submitted to the SAMPL7 physical property set of challenges. We report the following 6 error metrics: the root-mean-squared error (RMSE), mean absolute error (MAE), mean (signed) error (ME), coefficient of determination (R^2^), linear regression slope (m), and Kendall’s Tau rank correlation coefficient ($\tau$). Additionally, 95% confidence intervals were computed for these values using a bootstrapping-over-molecules procedure (with 10,000 bootstrap samples), as in prior SAMPL challenges [12].

Accuracy based performance metrics, such as RMSE and MAE, are more appropriate than correlation-based statistics to evaluate methods because of the small dynamic range of experimental log *P* values (0.6–3.0). This is usually reflected in the confidence intervals on these metrics. Calculated error statistics of all methods can be found in Tables S1,  S3, and  S4. Summary statistics were calculated for each submission for method comparison. Details of the analysis and scripts are preserved on the SAMPL7 GitHub repository (described in the “Code and data availability” section).

For each challenge we included a reference and/or null method set of predictions in the analysis to provide perspective for performance evaluations of blind predictions. Null models or null predictions employ a model that is not expected to be useful and can provide a simple point of comparison for more sophisticated methods, as ideally, such methods should improve on predictions from a null model. Reference methods are not formally part of the challenge, but are provided as comparison methods. For the log *P* challenge we included a null prediction set which predicts a constant log *P* value of 2.66 for every compound, as described in a previous SAMPL paper [38]. For log *D* evaluation we included a set of null predictions that all of the molecules partition equally between the water and octanol phase.

For the log *P* and p*K*_a_ challenge and the log *D* evaluation, we provide reference calculations using ChemAxon’s Chemicalize [80], a commercially available empirical toolkit, as a point of comparison. These include *REF#* in the method name in all of the figures so that they are easily recognized as non-blind reference calculations. The analysis is presented with and without the inclusion of reference and/or null calculations in the SAMPL7 GitHub repository. The figures and statistics tables pertaining to the log *P* and p*K*_a_ challenges and the log *D* evaluation in this manuscript include reference calculations.

For the log *P* and p*K*_a_ challenge, we list consistently well-performing methods that were ranked in the top consistently according to two error and two correlation metrics: RMSE, MAE, R^2^, and Kendall’s Tau. These are shown in Table 2 and 4.

For each challenge, we also evaluated the relative difficulty of predicting the physical property of interest of each molecule in the set. We plotted the distributions of errors in prediction for each molecule considering all prediction methods. We also calculated the MAE for each molecule as an average of all methods, as well as for predictions from each method category.

#### Converting relative free energies between microstates to macroscopic p*K*_a_

In the p*K*_a_ challenge, participants submitted predictions consisting of the free energy changes between a reference microstate and every other relevant microstate for each compound. Specifically, participants were asked to predict the relative free energy between a selected neutral reference microstate and the rest of the enumerated microstates for that molecule at a reference pH of 0. In order to compare participants’ predictions to experimental p*K*_a_ values, these predicted relative free energies had to be converted to macroscopic p*K*_a_ values.

**Fig. 3:**
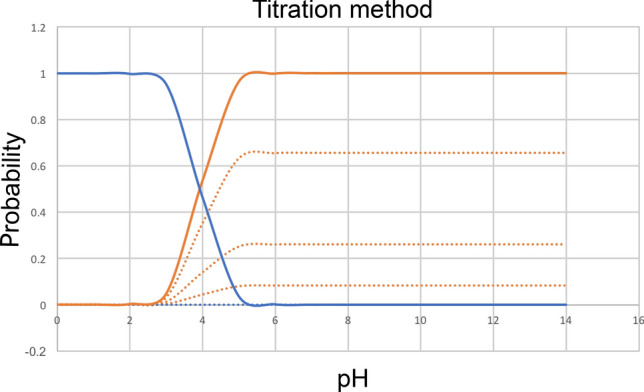
Using the microstate probability to convert microscopic p*K*_a_ predictions to macroscopic p*K*_a_’s with the titration method p*K*_a_’s. Blue and orange lines represent two states. Blue states have one more proton than the orange states, and thus a formal charge higher by + 1. The blue state has one tautomer and the orange state has 3, denoted by the dashed lines. The solid lines are the ensemble averaged state probability for each group with a given charge. The crossing point between two ensemble lines is the macroscopic p*K*_a_

Here, we analyzed submissions using the titration method discussed above (Approaches to predicting small molecule p*K*_a_’s sect.). This approach computes the population of each charge state as a function of pH and finds the pH at which the population of one charge state crosses that of another (Figure 3); as noted above this approach is equivalent to the transition and free energy approaches detailed previously.

In our analysis Python code used in the present challenge we work from Eqs. 6 and 7 to find the pH at which populations of the two charge states are equal. Here, we do this using fsolve from scipy in Python.

## Results and discussion

### Overview of log *P* challenge results

A variety of methods were used in the log *P* challenge. There were 33 blind submissions collected from 17 groups (Tables of participants and their predictions can be found in the SAMPL7 GitHub Repository and in the Supporting Information.). In the SAMPL6 octanol-water log *P* challenge there were 91 blind submissions collected from 27 participating groups. In the SAMPL5 Cyclohexane-Water log *D* challenge, there were 76 submissions from 18 participating groups [34], so participation was lower than previous iterations. This modestly decreased participation (by one group) was likely in part because of COVID-19-related disruptions and because this challenge had to be conducted on a short timescale with relatively limited publicity because the experimental data was not generated specifically for SAMPL, and thus staging of the SAMPL7 challenge required delaying submission of an experimental study which was already complete.

Out of blind submissions of the SAMPL7 log *P* challenge, there were 10 in the physical (MM) category, 10 in the physical (QM) category, and 12 in the empirical category An additional null and reference method were included in the empirical method category.

The following sections evaluate the performance of log *P* prediction methods. Performance statistics of all the methods can be found in Table S1. Methods are referred to by their method names, which are provided in Table 1.

#### Performance statistics to compare log *P* prediction methods

**Fig. 4:**
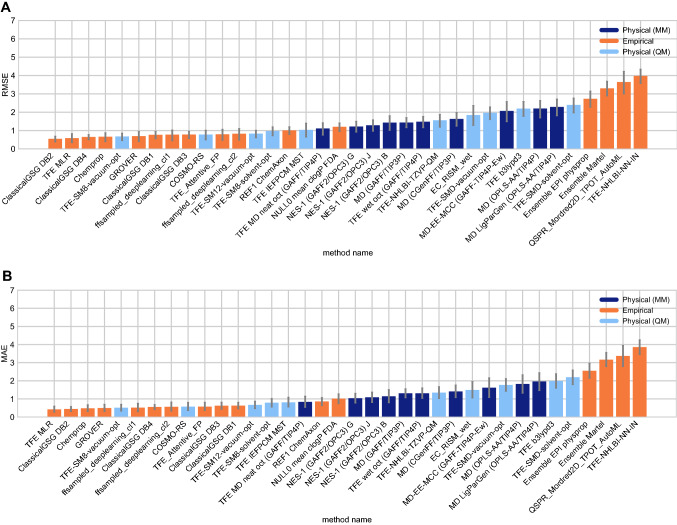
Overall accuracy assessment for all methods participating in the SAMPL7 log *P* challenge shows that many methods did not exhibit statistically significant differences in performance and there was no single clear winner; however, empirical methods tended to perform better in general. Both root-mean-square error (RMSE) and mean absolute error (MAE) are shown, with error bars denoting 95% confidence intervals obtained by bootstrapping over challenge molecules. Empirical methods outperform the majority of the other methods. Methods that achieved a RMSE $\le$ 1.0 log *P* units were mainly empirical based, and some were QM-based physical methods. Submitted methods are listed in Table 1. The submission *REF1 ChemAxon* [80] was a reference method included after the blind challenge submission deadline, and *NULL0 mean cLogP FDA* is the null prediction method; all others refer to blind predictions

Some methods in the challenge achieved a good octanol–water log *P* prediction accuracy. Figure 4 shows the performance comparison of methods based on accuracy with RMSE and MAE. The uncertainty in the correlation statistics was too high to rank method performance based on correlation, but we provide an overall correlation assessment for all methods in the SI in Figure S2. 16 submissions achieved a RMSE $\le$ 1.0 log *P* units, but no method achieved a RMSE $\le$ 0.5 log *P* units. Methods that achieved a RMSE $\le$ 1.0 log *P* units were mainly empirical, but some were QM-based. Prediction methods include 15 blind predictions and one reference method.

#### A shortlist of consistently well-performing methods in the log *P* challenge

Here, many performance differences are not statistically significant, but we identified five consistently well-performing ranked methods that appear in the top 10 according to two accuracy based (RMSE and MAE) and two correlation based metrics (Kendall’s Tau and R^2^), as shown in Table 2. The resulting 5 best-performing methods were made up of three empirical methods and two QM-based physical methods.

**Table 2:** Five consistently well-performing log *P* prediction methods based on consistent ranking within the top 10 according to various statistical metrics

Method name	Category	RMSE	MAE	R^2^	Kendall’s Tau
*TFE MLR* [87]	Empirical	0.58 [0.34, 0.83]	0.41 [0.26, 0.60]	0.43 [0.06, 0.80]	0.56 [0.23, 0.83]
*Chemprop* [88]	Empirical	0.66 [0.39, 0.89]	0.48 [0.30, 0.69]	0.41 [0.11, 0.76]	0.54 [0.25, 0.82]
*ClassicalGSG DB3* [84–86]	Empirical	0.77 [0.57, 0.96]	0.62 [0.43, 0.82]	0.51 [0.18, 0.77]	0.48 [0.14, 0.75]
*COSMO-RS* [89]	Physical (QM)	0.78 [0.49, 1.01]	0.57 [0.36, 0.80]	0.49 [0.17, 0.80]	0.53 [0.25, 0.78]
*TFE-NHLBI-TZVP-QM*	Physical (QM)	1.55 [1.19, 1.87]	1.34 [1.02, 1.76]	0.52 [0.19, 0.78]	0.51 [0.19, 0.78]

**Fig. 5:**
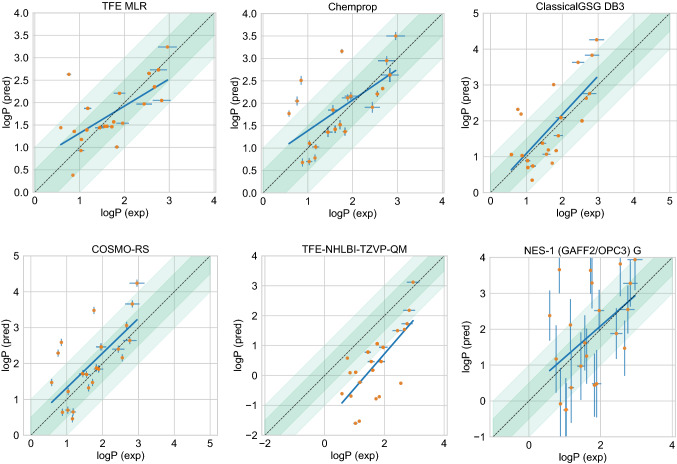
Predicted vs. experimental value correlation plots of 5 best performing methods and one representative average method in the SAMPL7 log *P* challenge. Dark and light green shaded areas indicate 0.5 and 1.0 units of error. Error bars indicate standard error of the mean of predicted and experimental values. In some cases, log *P* SEM values are too small to be seen under the data points. The best-performing methods were made up of three empirical methods (*ClassicalGSG DB3* [85], *TFE MLR* [87], *Chemprop* [88]) and two QM-based physical methods (*COSMO-RS* [89], *TFE-NHLBI-TZVP-QM*). Details of the methods can be found in "A shortlist of consistently well-performing methods in the p*K*_a_ challenge" sect. and performance statistics are available in 2. Method *NES-1 (GAFF2/OPC3 G)* was selected as the representative average method, which has a median RMSE

Submissions were ranked according to RMSE, MAE, R^2^, and Kendall’s Tau. Many top methods were found to be statistically indistinguishable when considering the uncertainties of their error metrics. Additionally, the sorting of methods was significantly influenced by the metric that was chosen. We determined which ranked log *P* prediction methods were consistently the best according to all four chosen statistical metrics by assessing the top 10 methods according to each metric. A set of five consistently well-performing methods were determined– three empirical methods and two QM-based physical methods. Performance statistics are provided as mean and 95% confidence intervals. Correlation plots of the best performing methods and one average method is shown in Figure 5. Additional statistics are available in Table S1

Method *TFE MLR* [87] was an empirical method that used a multi-linear regression (MLR) made from experimental log *P* values from 60 sulfonamides obtained from PubChem [95] and DrugBank [96]. The dataset was mainly composed of sulfonamide drugs and smaller molecules with other classical functional groups. The following descriptors were used to create the MLR: the frequency of functional groups, hydrogen bond acceptors, hydrogen bond donors, molar refractivity, and topological polar surface area. The functional group frequency was calculated with an in-house script from a modified function of Open Babel [97], the rest was obtained from supplied Open Babel properties.

Method *Chemprop* was an empirical method which used the log *P* dataset of the OPERA models in their approach [88]. Molecules from the Opera set were compared with the challenge molecules and those with an ECFP_6 fingerprint (extended connectivity fingerprint) tanimoto coefficient (TC) greater than 0.25 were flagged as test molecules for a total of 233 testing molecules. The training set was created from the rest of the Opera data set by filtering out molecules with a ECFP_6 TC > 0.4 to test set molecules. Several models were built using a Directed-Message Passing Neural Network (D-MPNN) [98, 99] to predict the log *P*, which was then used to get the transfer free energy.

Submission *ClassicalGSG DB3* is an empirical method that employed neural networks (NNs) where the inputs are molecular features generated using a method called Geometric Scattering for Graphs (GSG) [84–86]. In GSG, atomic features are transformed into molecular features using the graph molecular structure. For atomic features, predictions used 4 physical quantities from classical molecular dynamics forcefields: partial charge, Lennard-Jones well depth, Lennard-Jones radius and atomic type. A training dataset was built from 7 datasets for a total of 44,595 unique molecules. Open Babel was used to convert RDKit generated canonical SMILES to MOL2 files, which were then used as input into CGenFF to determine partial charges and Lennard-Jones parameters for all atoms in each molecule. The generation of CGenFF atomic attributes failed for some molecules, so the final dataset had 41,409 molecules, and is referred to as the “full dataset”. A training set of 2379 molecules was obtained by filtering the full training set and keeping only those with sulfonyl functional groups. This was done using the HasSubstructMatch function of the RDKit toolkit. The log *P* values were predicted by the model trained on this training set.

Method *COSMO-RS* was a QM-based physical prediction approach [89].. First, this approach used COSMOquick [100] to generate tautomers and discarded irrelevant states due to an internal energy threshold implemented in COSMOquick. The participants conducted a conformational search of every microstate with COSMOconf [101] using up to 150 conformers. Second, for each conformer they performed a geometry optimization using the BP86 functional with a TZVP basis set and the COSMO solvation scheme, followed by a single point energy calculation using the BP86 functional with a def2-TZVPD basis set and the FINE COSMO cavity. All density functional theory calculations were carried out with the TURBOMOLE 7.5 program package [102, 103]. Third, a conformer selection was done by applying COSMOconf (using internally COSMOtherm) to reduce the number of conformers and tautomers for the neutral molecule sets. The final set of the neutral state contained only those conformers and states that are relevant in liquid solutions. Fourth, the COSMOtherm software (version 2020) [104] was used to calculate the free energy difference for each molecule set (from the second step described here) and to calculate the relative weight of the microstates in water. All free energy calculations were carried out using the BP-TZVPD-FINE 20 level of COSMO-RS in COSMOtherm. Within the used COSMO-RS, an ensemble of conformers and microstates is automatically used and weighted according to the total free energy in the respective liquid phase, i.e. different weights are used in water and octanol.

Submission *TFE-NHLBI-TZVP-QM* was a QM-based physical method that used the Def2-TZVP basis set for all calculations. Calculations were performed in either Gaussian 09 or Gaussian 16. Structures were optimized with the B3LYP density functional and were verified to be local minima via frequency calculations on an integration grid with harmonic frequencies. Details of solvation handling were not included in the method description.

Figure 5 show predicted log *P* vs experimental log *P* value comparison plots of these 5 well-performing methods and also a method that represents average performance in this challenge. Representative method *NES-1 (GAFF2/OPC3) G* was selected because it has the median RMSE of all ranked methods analyzed in the challenge.

#### Difficult chemical properties for log *P* predictions

**Fig. 6:**
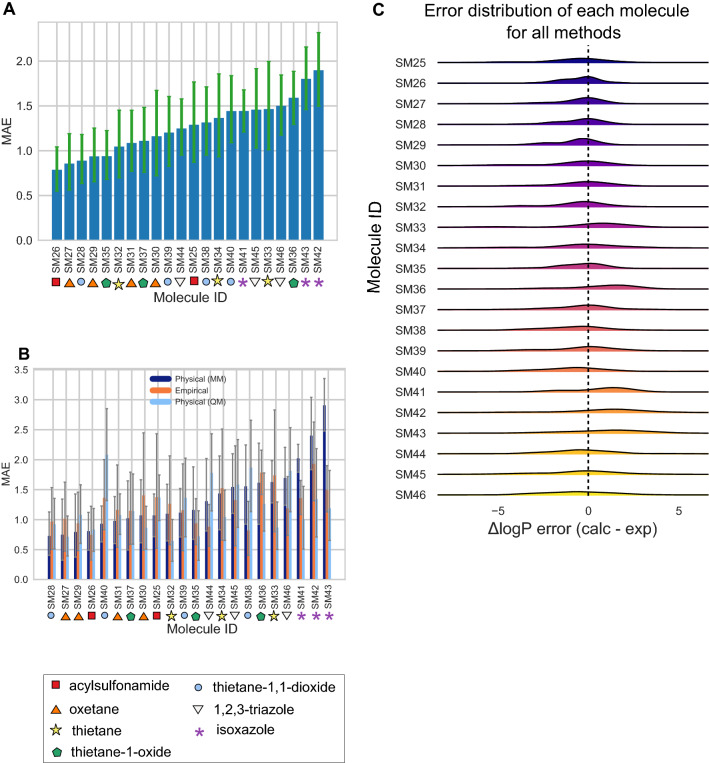
Molecule-wise prediction accuracy in the log *P* challenge point to isoxazoles as poorly predicted, especially by MM-based physical methods. Molecules are labeled with their compound class as a reference. **A** The MAE calculated for each molecule as an average of all methods. **B** The MAE of each molecule separated by method category. **C** log *P* prediction error distribution for each molecule across all prediction methods

To learn about chemical properties that are challenging for log *P* predictions, we analyzed the prediction errors of the molecules (Figure 6). We chose to use MAE for this analysis because it is less affected by outliers compared to RMSE and is therefore more appropriate for following global trends. Although methods varied in performance, as indicated by large and overlapping confidence intervals, the MAE calculated for each molecule as an average across all methods indicates that some of the molecules were better predicted than others (Figure 6A). For reference, compound classes and structures of the molecules are available in Figure S3. Molecules such as SM26, SM27, and SM28 were well predicted on average. Molecules such as SM42, SM43, and SM36 were not well predicted on average.

Certain groups of molecules seem to be more challenging for log *P* predictions. Two of the most poorly predicted molecules, SM42 and SM43, are isoxazoles. Isoxazoles are oxygen and nitrogen-containing heteroaromatics. When we consider the calculated MAE of each molecule separated out by method category, we find that predictions for 2 out of the 3 molecules (SM41 and SM43) belonging to the isoxazole compound class are less accurate with MM-based physical methods than with QM-based physical and empirical method categories (Figure 6B).

Figure 6C shows error distribution for each challenge molecule over all prediction methods. Molecules such as SM33, SM36, SM41, SM42, and SM43 are shifted to the right, indicating that methods likely had a tendency to overestimate how much these molecules favored the octanol phase.

### Overview of p*K*_a_ challenge results

In the SAMPL7 p*K*_a_ challenge there were 9 blind submissions from 7 different groups. Blind submissions included 7 QM-based physical methods, 1 QM+LEC method, and 1 QSPR/ML method. An additional reference prediction method was included in the QSPR/ML method category.

#### p*K*_a_ performance statistics for method comparison

**Fig. 7:**
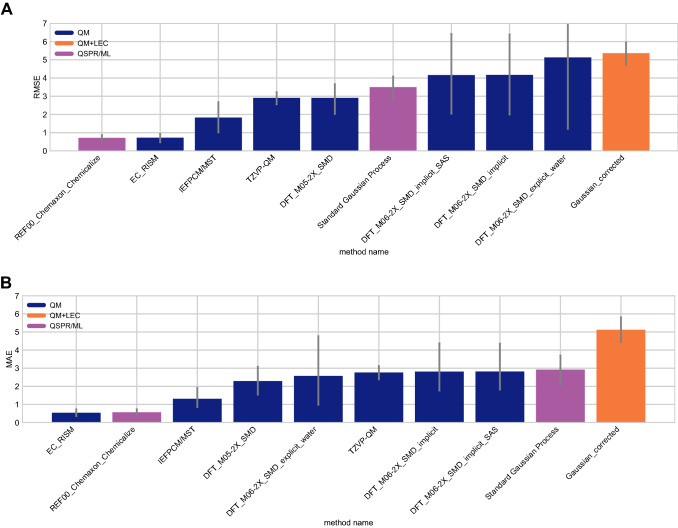
Overall accuracy assessment for all methods participating in the SAMPL7 p*K*_a_ challenge shows that two methods, one a Physical (QM) method and one a QSPR/ML, performed better than other methods. Both root-mean-square error (RMSE) and mean absolute error (MAE) are shown, with error bars denoting 95% confidence intervals obtained by bootstrapping over challenge molecules. *REF00_Chemaxon_Chemicalize* [80] is a reference method that was included after the blind challenge submission deadline, and all other method names refer to blind predictions. Methods are listed out in Table 3 and statistics calculated for all methods are available in Table S3

Some methods in the SAMPL7 challenge achieved a good prediction accuracy for p*K*_a_’s. Figure 7 shows the performance comparison of methods based on accuracy with RMSE and MAE. Two submissions achieved a RMSE < 1.0 p*K*_a_ units, no methods achieved a RMSE $\le$ 0.5 p*K*_a_ units. One of the methods that achieved a RMSE $< 1.0$ p*K*_a_ units was a QM-based physical prediction method (*EC_RISM* [92]), and the other was a QSPR/ML method that was submitted as a reference method (*REF00_Chemaxon_Chemicalize* [80]).

**Fig. 8:**
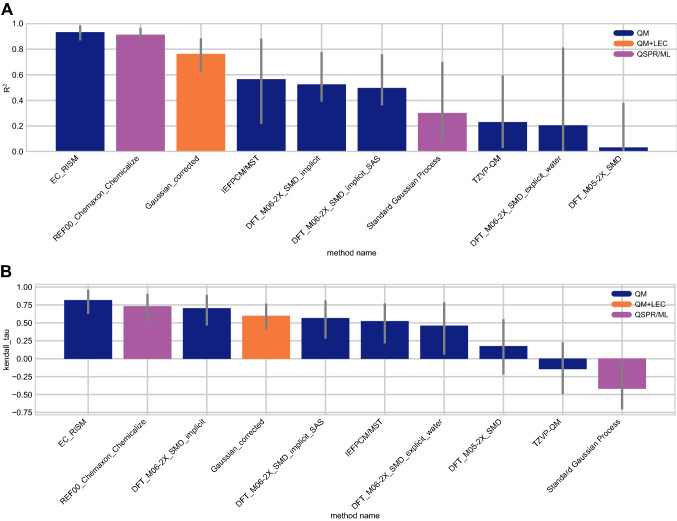
Overall correlation assessment for all methods participating in the SAMPL7 p*K*_a_ challenge shows that one Physical (QM) method and one QSPR/ML reference method exhibited modestly better performance than others. Pearson’s R^2^ and Kendall’s Rank Correlation Coefficient Tau ($\tau$) are shown, with error bars denoting 95% confidence intervals obtained by bootstrapping over challenge molecules. Submission methods are listed out in Table 3. *REF00_Chemaxon_Chemicalize* [80] is a reference method that was included after the blind challenge submission deadline, and all other method names refer to blind predictions. Most methods have a statistically indistinguishable performance on ranking, however, for R^2^, two methods (*EC_RISM* [92], *REF_Chemaxon_Chemicalize*), tend to have a greater ranking ability than the other methods. Evaluation statistics calculated for all methods are available in Table S3 of the Supplementary Information

Correlation-based statistics methods provide a rough comparison of methods. Figure 8 shows R^2^ and Kendall’s Tau values calculated for each method, sorted from high to low performance. It is not possible to truly rank these methods based on correlation due to the high uncertainty of each correlation statistic. Over half of the methods have R^2^ and Kendall’s Tau values equal to or greater than 0.5 and can be considered as the better half, however individual performance is largely indistinguishable from one another. For R^2^, two methods (*EC_RISM*, *REF00_Chemaxon_Chemicalize*), seem to have a greater ranking ability than the other methods.

There were six methods with an R^2^ $\ge$ 0.5— four of the methods were QM methods, one was a QM+LEC method, and one was a QSPR/ML method. Seven methods had a Kendall’s Tau $\ge$ 0.50. Of these, five were QM methods, one was a QM+LEC method, and one was a QSPR/ML method.

**Table 3:** Method names, category, and submission type for all the p*K*_a_ submissions. The “submission type” column indicates if submission was a blind submission (denoted by “Blind”) or a post-deadline reference calculation (denoted by “Reference”). The table is ordered from lowest to highest RMSE, although many consecutively listed methods are statistically indistinguishable. All calculated error statistics are available in Table S3

Method name	Category	Submission type
*REF00_Chemaxon_Chemicalize* [80]	QSPR/ML	Reference
*EC_RISM* [92]	QM	Blind
*IEFPCM/MST* [90]	QM	Blind
*DFT_M05-2X_SMD* [94]	QM	Blind
*TZVP-QM*	QM	Blind
*Standard Gaussian Process*	QSPR/ML	Blind
*DFT_M06-2X_SMD_implicit*	QM	Blind
*DFT_M06-2X_SMD_implicit_SAS*	QM	Blind
*DFT_M06-2X_SMD_explicit_water*	QM	Blind
*Gaussian_corrected*	QM+LEC	Blind

#### A shortlist of consistently well-performing methods in the p*K*_a_ challenge

We determined a group of consistently well-performing methods in the p*K*_a_ challenge. When looking at individual error metrics, many submissions are not different from one another in a way that is statistically significant. Ranking among methods changes based on the chosen statistical metric and does not necessarily lead to strong conclusions due to confidence intervals that often overlap with one another. Here, we determined consistently well-performing methods according to two accuracy (RMSE and MAE) and two correlation metrics (Kendall’s Tau and R^2^). For ranked submissions, we identified two consistently well-performing methods that were ranked in the top three according to these statistical metrics. The list of consistently well-performing methods are presented in Table 4. The resulting two best-performing methods were both QM-based physical methods.

**Table 4:** Two consistently well-performing p*K*_a_ prediction methods based on consistent ranking within the top three according to various statistical metrics

Method name	Category	RMSE	MAE	R^2^	Kendall’s Tau
*EC_RISM* [92]	QM	0.72 [0.45, 0.95]	0.53 [0.33, 0.75]	0.93 [0.87, 0.98]	0.81 [0.63, 0.96]
*IEFPCM/MST* [90]	QM	1.82 [1.00, 2.69]	1.30 [0.84, 1.92]	0.56 [0.22, 0.87]	0.52 [0.22, 0.76]

Ranked submissions were ranked/ordered according to RMSE, MAE, R^2^, and Kendall’s Tau. Many methods were found to be statistically indistinguishable when considering the uncertainties of their error metrics. Additionally, the sorting of methods was significantly influenced by the metric that was chosen. We determined which methods are repeatedly among the top two according to all four chosen statistical metrics by assessing the top three methods according to each metric. Two QM-based methods consistently performed better than others. Performance statistics are provided as mean and 95% confidence intervals. All statistics for all methods are in Table S3

Submission *EC_RISM* was a QM-based physical method [92]. In this approach, multiple geometries were generated for each microstate using the EmbedMultipleConfs function of RDKit. These structures were pre-optimized with Amber 12 using GAFF 1.7 parameters and AM1-BCC charges with an ALPB model to represent the dielectric environment of water. Conformations with an energy of more than 20 kcal/mol than the minimum structure of that microstate were discarded and the remaining structures clustered with a structural RMSD of 0.5 Angstrom. The cluster representatives were then optimized using Gaussian 16revC01 with IEF-PCM using default settings for water at the B3LYP/6-311+G(d,p) level of theory. Additional stereoisomers were treated as if they were additional conformational states of the same microstate so that for each microsate only up to 5 conformations with the lowest PCM energies for each solvent were treated with EC-RISM/MP2/6-311+G(d,p) using the PSE2 closure [54] and the resulting EC-RISM energies were corrected. To calculate the relative free energies with respect to each neutral reference state, 4 different formulas were used, depending on the difference in the protonation state. Macrostate p*K*_a_ values were calculated using the partition function approach of equation 5 found elsewhere [54].

Submission *IEFPCM/MST* was a QM-based physical method [90]. This approach used the Frog 2.14 software [105, 106] to explore microstate conformations. The molecular geometries of the compounds were fully optimized at the B3LYP/6-31G(d) level of theory, taking into account the solvation effect of water on the geometrical parameters of the solutes, using the IEFPCM version of the MST model. The resulting minima were verified by vibrational frequency analysis, which gave positive frequencies in all cases. The relative energies of the whole set of conformational species were refined from single-point computations performed at the MP2/aug-cc-pVDZ levels of theory. In addition, the gas phase estimate of the free energy difference for all microstates was derived by combining the MP2 energies with zero point energy corrections. Finally, solvation effects were added by using the B3LYP/6-31G(d) version of the IEFPCM/MST model, which is a quantum mechanical self-consistent continuum solvation method. The p*K*_a_ was determined using both the experimental hydration free energy of the proton (-270.28 kcal/mol) and a Boltzmann’s weighting scheme to the relative stabilities of the conformational species determined for the microstates involved in the equilibrium constant for the dissociation reaction following the thermodynamic cycle reported in previous studies [107].

**Fig. 9:**
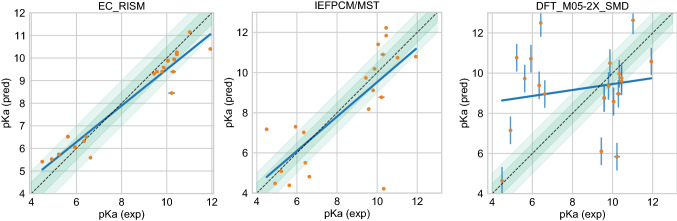
Predicted vs. experimental value correlation plots of 2 best performing methods and one representative average method in the SAMPL7 p*K*_a_ challenge. Dark and light green shaded areas indicate 0.5 and 1.0 units of error. Error bars indicate standard error of the mean of predicted and experimental values. Some SEM values are too small to be seen under the data points. Method *DFT_M05-2X_SMD* [94] was selected as the method with the median RMSE of all ranked methods analyzed in the challenge. Performance statistics of these methods is available in Table 4

Figure 9 show predicted p*K*_a_ vs experimental p*K*_a_ value comparison plots of the two well-performing methods and also a method that represents average performance. Representative average method *DFT_M05-2X_SMD* [94] was selected as the method with the median RMSE of all ranked methods analyzed in the challenge.

#### Difficult chemical properties for p*K*_a_ predictions

**Fig. 10:**
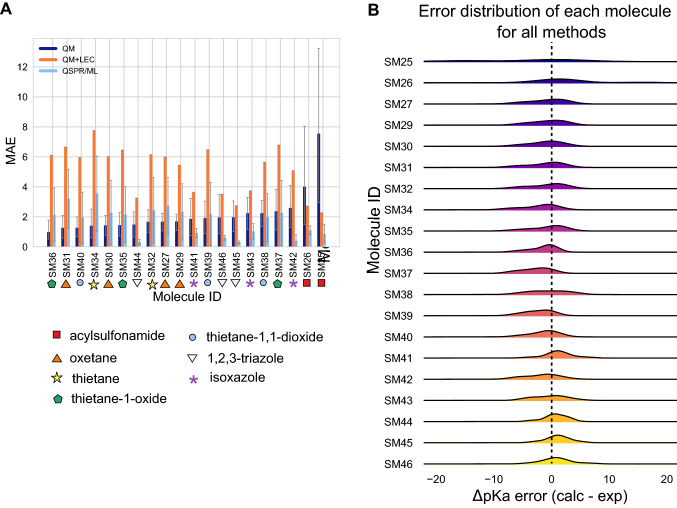
Molecule-wise prediction error distribution plots show the prediction accuracy for individual molecules across all prediction methods for the p*K*_a_ challenge. Molecules are labeled with their compound class as a reference. **A** The MAE of each molecule separated by method category suggests the most challenging molecules were different for each method category. It is difficult to draw statistically significant conclusions where there are large overlapping confidence intervals. The QM+LEC method category appears to be less accurate for the majority of the molecules compared to the other method categories. QSPR/ML methods performed better for isoxazoles (SM41-SM43) and 1,2,3-triazoles (SM44-SM46) compared to the other two method categories. Physical QM-based methods performed poorly for acylsulfonamides (SM26 and SM25). **B** Error distribution for each molecule over all prediction methods. SM25 has the most spread in p*K*_a_ prediction error

To learn about chemical properties that pose challenges for p*K*_a_ predictions, we analyzed the prediction errors of the molecules (Figure 10). For reference, compound classes and structures of the molecules are available in Figure S3. We chose to use MAE for molecular analysis because it is less affected by outliers compared to RMSE and is, therefore, more appropriate for following global trends. When we consider the calculated MAE of each molecule separated out by method category the prediction accuracy of each molecule varies based on method category (Figure 10A). The MAE calculated for each molecule as an average of all methods shows that SM25 was the most poorly predicted molecule. The QM+LEC method category appears to be less accurate for the majority of the molecules compared to the other method categories. Compared to the other two method categories, QSPR/ML methods performed better for molecules SM41–SM43, which are isoxazoles (oxygen and nitrogen containing heteroaromatics), and molecule SM44–SM46, which are 1,2,3-triazoles (nitrogen containing heteroaromatics). Physical QM methods performed poorly for molecules SM25 and SM26 (acylsulfonamide compound class). Figure 10B shows error distribution for each challenge molecule over all the prediction methods. Molecule SM25 has the most spread in p*K*_a_ prediction error.

#### Microscopic p*K*_a_ performance

SAMPL7 challenge p*K*_a_ participants were asked to report the relative free energy between microstates, using a provided neutral microstate as reference. Microstates are defined as the enumerated protomers and tautomers of a molecule. Details of how microstates were found can be found in "Microstate enumeration" sect. Some molecules had 2 microstates, while others had as many as 6 (Table S7).

Figure 12 shows the predicted free energy change between the reference state and each microstate, on average, for all transitions across all predictions. Molecules are labeled with their compound class as a reference. Predictions disagree widely for some transitions, like those from the reference state to SM26_micro002, SM28_micro001, SM43_micro003, SM46_micro003, while predictions for other transitions such as that from the reference microstate to SM26_micro004 are in agreement (as shown by small error bars in Figss. 12A, 14).

Figure 14 shows examples of some microstate transitions where participants’ predicted transition free energies disagree. We also examined how the microstate transition free energies (relative to the reference state) are distributed across predictions (Fig. 12B). We find that some transitions are much more consistently predicted than others, but in some cases there is broad disagreement even about the sign of the free energy change associated with the particular transition—so methods disagree as to which protonation state or tautomer is preferred at the reference pH.

To further analyze which transitions were difficult, we focused on how consistently methods agreed as to the sign of the free energy change for each transition. Particularly, we calculated the Shannon Entropy (H) for the transition *sign* for each transition, shown in Fig. 13. For each microstate, we calculated H via:

17 $$\begin{aligned} H = -\sum _i P_{i}ln\left( P_{i}\right) \end{aligned}$$

where P_i_ is the probability of a particular outcome *i*; here, we use *i* to indicate a positive sign or a negative sign for the predicted free energy change. So P_positive_ is the fraction of positive sign predictions, P_negative_ is the fraction of negative sign predictions, and P_neutral_ is the fraction of neutral sign predictions (which were somewhat frequent as a few participants predicted a free energy change of exactly 0 for some transitions). For example, for SM25_micro001, given the predictions we received, the P_positive_ is 0.5, the P_negative_ is 0.4 and the P_neutral_ is 0 (no neutral sign predictions). The Shannon entropy H is then $-(0.5 \ln (0.5)+0.4 \ln (0.4)+0)$, which is roughly 0.7 and indicates predictions had difficulty agreeing on the sign.

While the Shannon entropy may not be a perfect tool for analyzing this issue, we find it helpful here. For a particular transition, a value of 0 indicates all predictions agreed as to the sign of the free energy change (whether positive, negative, or neutral), while values greater than 0 reflect an increasing level of disagreement in the sign of the prediction. 32 of the microstates had a H value of 0, 21 had a values that ranged from 0.5 to 0.7, and 3 microstates had values greater than 0.9 (the highest level of disagreement). The 3 microstates with the most disagreement belong to the thietane-1-oxide compound class (one from SM35, one from SM36 and one from SM37).

**Fig. 11:**
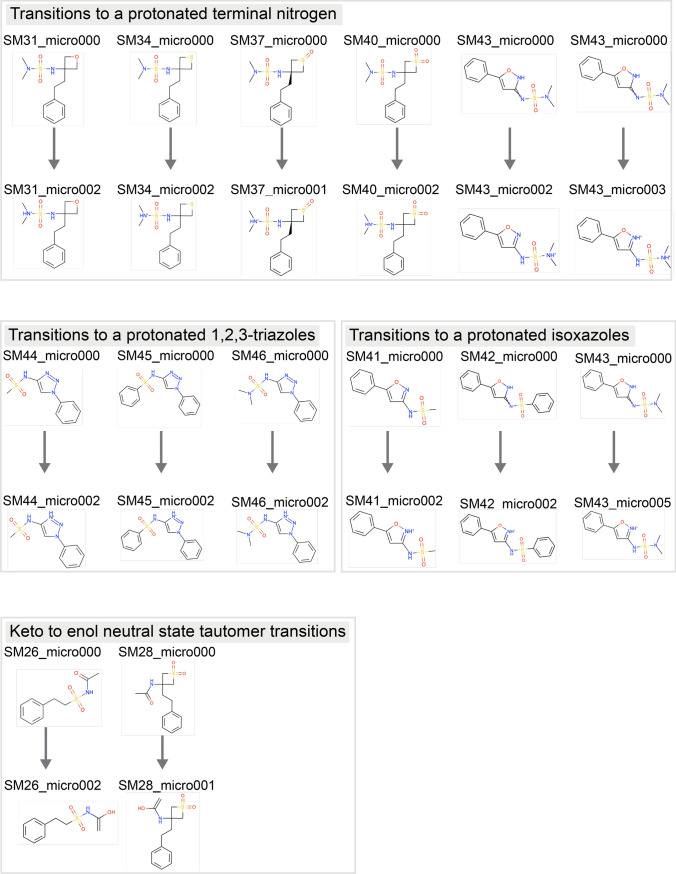
Chemical transformations that lead to common sign disagreements among participants typically involve a protonated nitrogen in terminal nitrogen groups, 1,2,3-triazoles, and isoxazoles. Shown are some chemical transformations that repeatedly show up as having large disagreement on the sign of the relative free energy prediction, as seen in Fig. 13

Transitions that pose difficulty for participants involve a protonated nitrogen and keto-enol neutral state tautomerism. Chemical transformations involving a protonated nitrogen in terminal nitrogen groups, 1,2,3-triazoles, and isoxazoles were all found to occur in molecules that have high levels of disagreement in sign prediction. Depictions of some of these types of transitions are presented in Fig. 11. Predictions for these transitions were substantially divided on the predicted sign—roughly half of the methods predict a positive sign, while the other half predict a negative sign. This means methods could not agree on the preferred state at the reference pH. The number of positive, negative, and neutral sign predictions per microstate is available in Table S5

In several cases, the SAMPL input files provided a reference microstate with unspecified stereochemistry, then a separate but otherwise equivalent microstate with specified stereochemistry (SM35_micro002, SM36_micro002, SM37_micro003). Experiments were done on the compound with specified stereochemistry, so participants were instructed to assume that the reference microstate (which had unspecified stereochemistry) had the same free energy as the microstate with specified stereochemistry. However, many participants didn’t use the microstate with specified stereochemistry as the reference state, and most ended up predicting a nonzero relative free energy between the reference state and the microstate with specified stereochemistry, despite instructions.

**Fig. 12:**
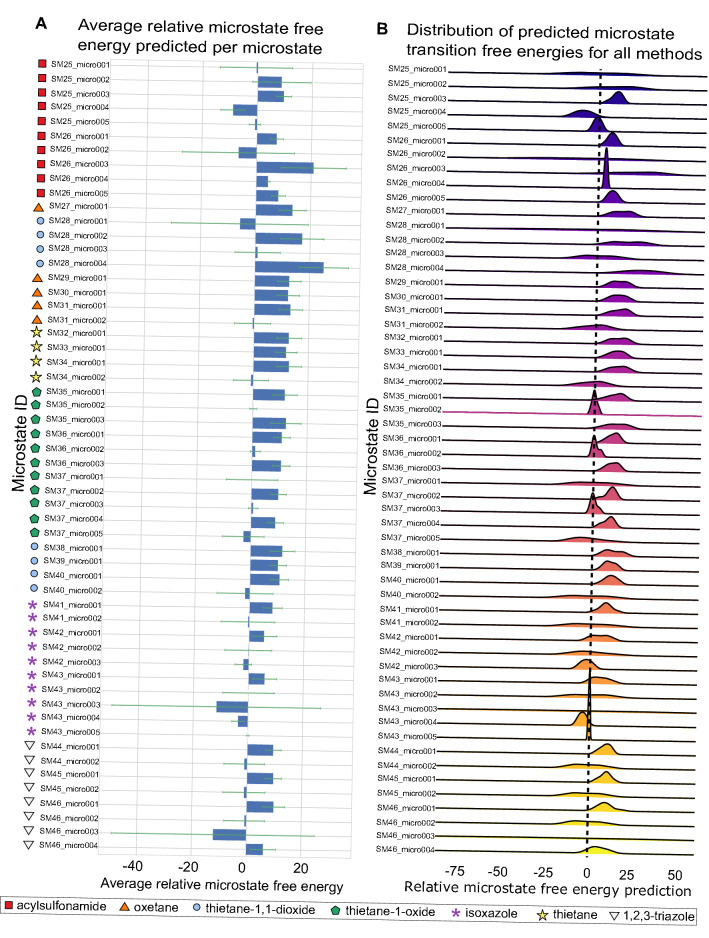
The average relative microstate free energy predicted per microstate and the distribution across predictions in the SAMPL7 p*K*_a_ challenge show how varied predictions were. Molecules are labeled with their compound class as a reference. **A** The average relative microstate free energy predicted per microstate. Error bars are the standard deviation of the relative microstate free energy predictions. A lower standard deviation indicates that predictions for a microstate generally agree, while a larger standard deviation means that predictions disagree. Predictions made for microstates such as SM25_micro001, SM26_micro002, SM28_micro001, SM43_micro003, SM46_micro003 widely disagree, while predictions for microstates such as SM26_micro004 are in agreement. **B** Distribution for each relative microstate free energy prediction over all prediction methods shows how prediction agreement among methods varied depending on the microstate

**Fig. 13:**
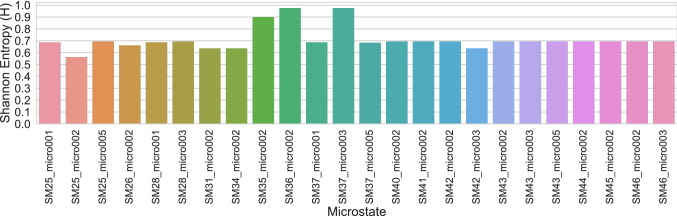
The Shannon entropy (H) per microstate transition shows that participants disagree on many of the signs of the relative free energy predictions. Microstates with entropy values greater than 0 reflect increasing disagreement in the predicted sign. Microstates with an entropy of 0 are not shown here, but indicate that methods made predictions which had the same sign for the free energy change associated with a particular transition. About 44% of all microstates predictions disagreed with one another based on the sign, and the rest agreed. Roughly 5% of microstates strongly disagreed on the sign of predictions—meaning that predicted relative free energies were fairly evenly split between positive, neutral, and negative values. This indicates that these transitions were particularly challenging

**Fig. 14:**
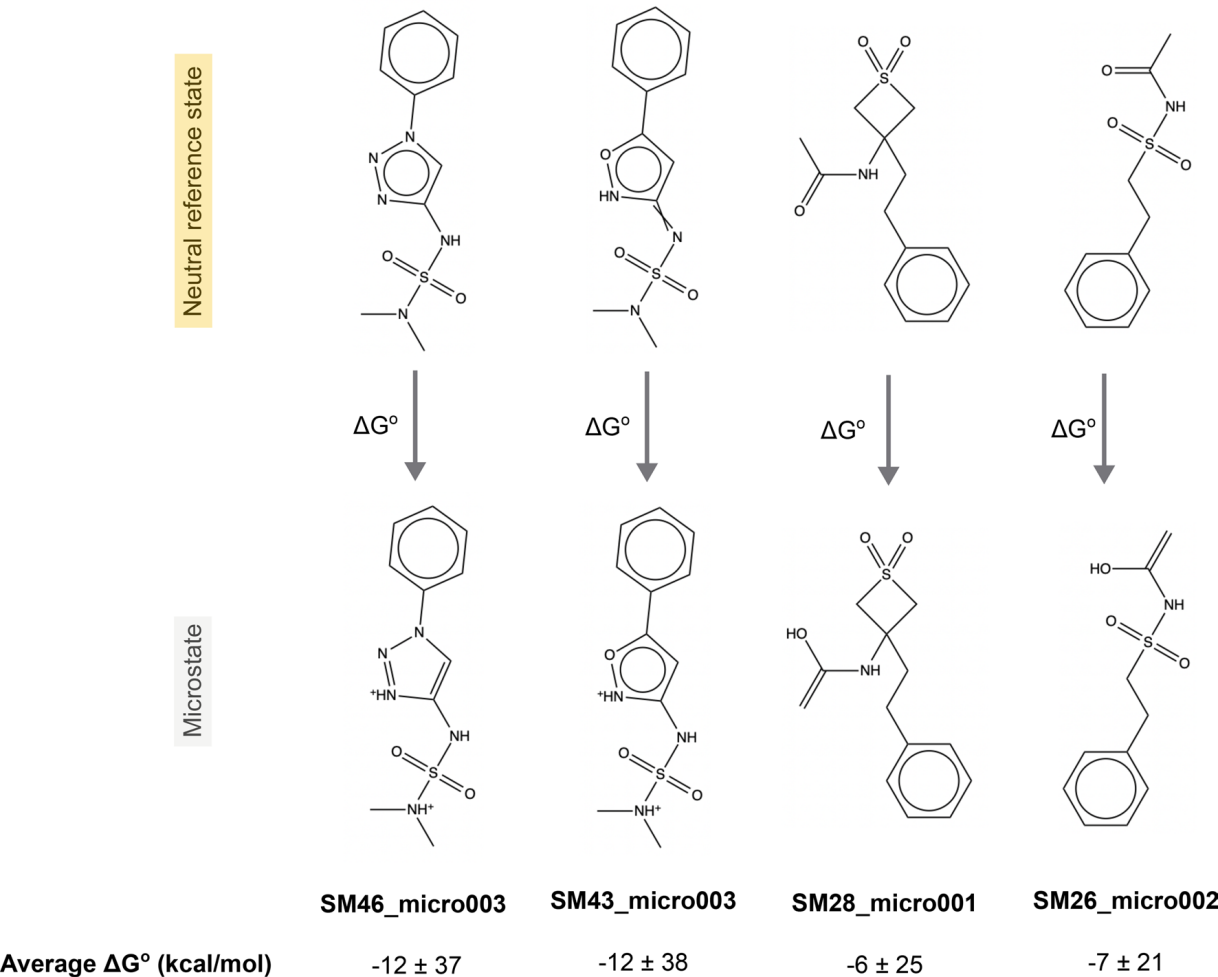
Structures of microstates where relative microstate free energy predictions disagree. Shown are some of the microstate transitions where participants predictions largely disagree with one another, based on Fig. 12. The average relative free energy prediction ($\Delta$G) along with the standard deviation are listed under each transition

### Overview of log *D* challenge results

In the SAMPL7 physical property prediction challenge, log *P* and p*K*_a_ predictions were combined in order to estimate log *D*, as described in "Combining log *P* and p*K*_a_ predictions to estimate log *D*" sect.

There were 6 log *D* estimates and 2 reference methods. Methods are listed in Table 5 and statistics for all log *D* prediction methods are available in Table S4. There were 5 methods that belonged to the physical (QM) category, and 1 in the Physical (MM) + QM+LEC category (this category used a MM-based physical method in the log *P* challenge, and a QM+LEC method in the p*K*_a_ challenge). The null and reference method were included in the empirical method category.

**Table 5:** Method names, category, and submission type for all the log *D* estimations

Method name	Category	Submission type
*REF0 ChemAxon*	Empirical	Reference
*TFE IEFPCM MST + IEFPCM/MST*	Physical (QM)	Standard
*NULL0*	Empirical	Reference
*EC_RISM_wet + EC_RISM*	Physical (QM)	Standard
*TFE-NHLBI-TZVP-QM + TZVP-QM*	Physical (QM)	Standard
*TFE b3lypd3 + DFT_M05-2X_SMD*	Physical (QM)	Standard
*MD (CGenFF/TIP3P) + Gaussian_corrected*	Physical (MM) + QM+LEC	Standard
*TFE-SMD-solvent-opt + DFT_M06-2X_SMD_explicit_water*	Physical (QM)	Standard

Method names are based off the submitted p*K*_a_ and log *P* method names, with the log *P* method name listed first followed by “+” and then the p*K*_a_ method name. The “submission type” column indicates if submission was a blind submission (denoted by “Blind”) or a post-deadline reference calculation (denoted by “Reference”). All calculated error statistics are available in Table S4

#### log *D* performance statistics for method comparison

**Fig. 15:**
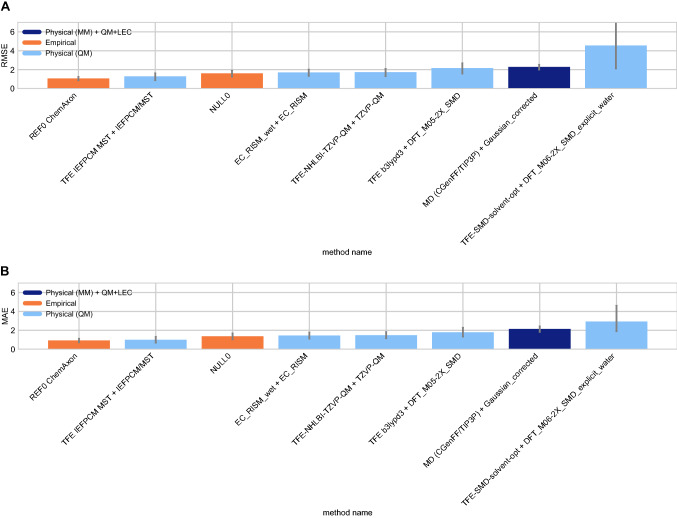
Overall accuracy assessment for log *D* estimation. Both root-mean-square error (RMSE) and mean absolute error (MAE) are shown, with error bars denoting 95% confidence intervals obtained by bootstrapping over challenge molecules. *REF00_ChemAxon* [80] is a reference method and *NULL0* is a null method that was included after the blind challenge submission deadline, and all other method names refer to blind predictions. Methods are listed out in Table 5 and statistics calculated for all methods are available in Table S4

**Fig. 16:**
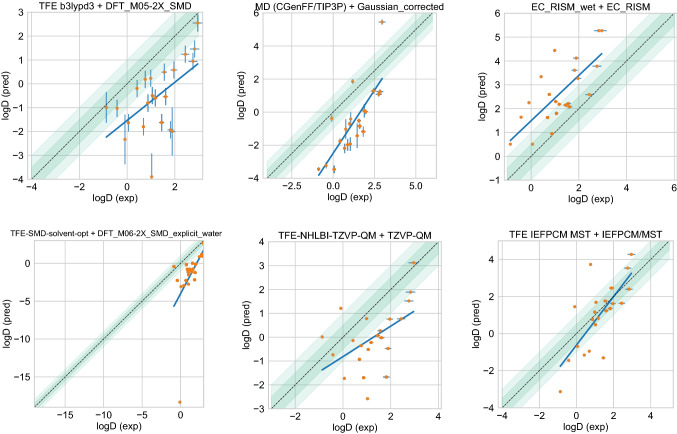
Predicted vs. experimental value correlation plots of all log *D* estimation methods in the SAMPL7 challenge. Dark and light green shaded areas indicate 0.5 and 1.0 units of error. Error bars indicate standard error of the mean of predicted and experimental values. Some SEM values are too small to be seen under the data points. Performance statistics of all methods is available in Table S4

Figure 15 compares the accuracy of methods based on RMSE and MAE. No method achieved a RMSE $\le$ 1.0 log *D* units, and the overall RMSE ranged from 1.1 to 4.5 log *D* units. Four methods had a RMSE between 1 and 2, and three methods had an RMSE between 2 and 3. Accuracy is better than the previous log *D* challenge. In the SAMPL5 log *D* challenge, out of 63 submissions, no submissions had a RMSE below 2 log *D* units. Here, eight methods were submitted and half of them achieved a RMSE below 2 log *D* units. Overall, log *D* prediction accuracy has improved since SAMPL5. Corresponding correlation plots are shown in Fig. 16.

**Fig. 17:**
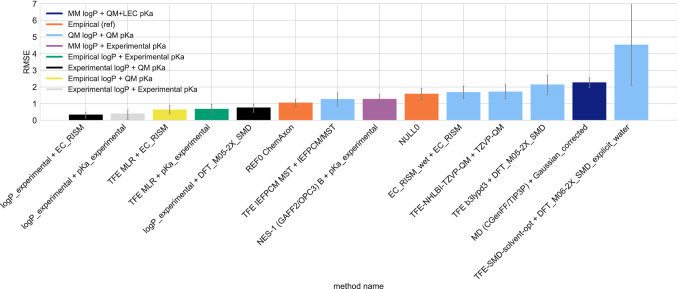
log *D* values from a combination of the best p*K*_a_ and log *P* are typically superior. Shown is the RMSE in calculated log *D* values, with error bars denoting 95% confidence intervals from bootstrapping over challenge molecules. This plot is similar to Fig. 4, except it includes some additional p*K*_a_ and log *P* combinations (for log *D* estimation). Method *logP_experimental + EC_RISM* combines the experimental log *P* with the top performing p*K*_a_ method (based on RMSE). Method *logP_experimental + pKa_experimental* combines the experimental log *P* and p*K*_a_ value. Method *TFE MLR + EC_RISM* combines the best performing (based on RMSE) log *P* and p*K*_a_ methods. Method *TFE MLR + pKa_experimental* combines the best performing (based on RMSE) log *P* method with the experimental p*K*_a_. Method *logP_experimental + DFT_M05-2X_SMD* combines the experimental log *P* with an average performing p*K*_a_ method. Method *NES-1 (GAFF2/OPC3) B + pKa_experimental* combines a log *P* method with average performance with the experimental p*K*_a_. All other methods are the same as in Fig. 4

When the best log *P* and p*K*_a_ prediction methods are combined we find that the resulting composite approach outperforms most of the other ranked methods, achieving a RMSE of 0.6 (see Figure 17, method name *TFE MLR + EC_RISM*).

When the experimental log *P* and p*K*_a_ are combined to yield a log *D* (as in "Combining log *P* and p*K*_a_ predictions to estimate log *D*" sect.), the resulting log *D* values do not perfectly match with the reported experimental log *D* values, an inconsistency that requires further investigation.

#### A consistently well performing method in log *D* estimation

For ranked submissions, we identified a single consistently well-performing method that was ranked in the top three according to RMSE, MAE, Kendall’s Tau, and R^2^ (all statistics are available in Table S4). The best-performing method was *TFE IEFPCM MST + IEFPCM/MST*, which used a QM-based physical method for p*K*_a_ and log *P* predictions [90]. The *IEFPCM/MST* model has previously been used to predict the log *D* of over 35 ionizable drugs, where it achieved a RMSE of 1.6 [108], all little worse than a RMSE of 1.3 in SAMPL7. The p*K*_a_ prediction protocol used in the challenge is described in "A shortlist of consistently well-performing methods in the log *P* challenge" sect., where it was ranked among the consistently well performing p*K*_a_ methods.

## Conclusions

Here, a community-wide blind prediction challenge was held that focused on partitioning and p*K*_a_ for 22 compounds composed of a series of N-acylsulfonamides and related bioisosteres. Participants had the option of submitting predictions for both, or either, challenge.

In the SAMPL7 log *P* challenge, participants were asked to predict a partition coefficient for each compound between octanol and water and report the result as a transfer free energy. A total of 17 research groups participated, submitting 33 blind submissions total. Many submissions achieved a RMSE around 1.0 or lower for log *P* predictions, but none were below 0.5 log *P* units. RMSEs ranged from 0.6 to 4 log *P* units—15 methods achieved a RMSE of 1.0 or lower, while a RMSE between 1 and 4 log units was observed for the majority of methods. Many methods achieved an accuracy similar to the null model which had a RMSE of 1.2 and predicted that each compound had a constant log *P* value of 2.66. A few methods outperformed the null model (4 were empirical and 1 was an QM based method). In general, empirical methods tended to perform better than other methods, which makes sense given the availability of octanol-water log *P* training data.

Performance in the SAMPL7 log *P* challenge was poorer than in the SAMPL6 log *P* challenge. In the SAMPL6 log *P* challenge, 10 methods achieved a RMSE $\le$ 0.5 log *P* units, while here, none did. In general, the SAMPL7 molecules were more flexible, which may have contributed to this accuracy difference. The chemical diversity in the SAMPL6 challenge dataset was limited to 6 molecules with 4-amino quinazoline groups and 2 molecules with a benzimidazole group. The SAMPL7 set was larger and more diverse, thus possibly more challenging.

For ranked submissions, we identified 5 consistently well-performing methods for log *P* evaluations based on several statistical metrics. These particularly well performing methods included three empirical methods, and two QM-based physical methods.

To see if any molecules posed particular challenges, we looked at log *P* prediction accuracy for each molecule across all methods. Compounds belonging to the isoxazole compound class had higher log *P* prediction errors. MM-based physical methods tended to make predictions that were less accurate for molecules belonging to the isoxazole compound class compared to QM-based physical and empirical method categories.

In the SAMPL7 p*K*_a_ challenge, participants predicted free energies for transitions between microstates. Predicted relative free energies were then converted to macroscopic p*K*_a_ values in order to compare participants’ predictions to experimental p*K*_a_ values and calculate performance statistics of predictions. This format allowed us to avoid some of the challenges of matching microscopic transitions to macroscopic p*K*_a_ values [43], making analysis more straightforward. As noted above, some matching is still required, but this approach eliminates uncertainty about which transitions are predicted.

Macroscopic p*K*_a_ evaluations relied on accuracy and correlation metrics. No method achieved a RMSE around 0.5 or lower for macroscopic p*K*_a_ predictions for the challenge molecules which means methods did not achieve experimental accuracy, which is likely around 0.5 p*K*_a_ units [109]. Methods had RMSE values between 0.7 to 5.4 p*K*_a_ units. Compared to the previous SAMPL6 p*K*_a_ challenge, accuracy remains roughly the same. Out of all submitted methods in SAMPL7, two methods achieved a RMSE lower than 1 p*K*_a_ unit (one of which was a commercially available method that we used as a reference method), while a RMSE between 1.8 and 5.4 log units was observed for the majority of methods. In terms of correlation, predictions had R^2^ values ranging from 0.03 to 0.93 and only two methods achieved an R^2^ greater than 0.9.

We tested ChemAxon’s Chemicalize toolkit [80] as an empirical reference method to make macroscopic p*K*_a_ predictions and it performed better than other methods. Excluding the reference method, the two best performing methods across several performance statistics were both QM-based physical methods.

For microscopic p*K*_a_, we find that some transitions are much more consistently predicted than others, but in some cases there is broad disagreement even about the sign of the free energy change associated with a particular transition—so methods disagree as to which protonation state or tautomer is preferred at the reference pH. Participants agreed on the sign of predictions for roughly 56% of all microstates, while 38% disagreed on sign (predictions were negative or positive). Certain chemical transformations were found to have a high level of disagreement, especially protonation of nitrogens in 1,2,3-triazoles, isoxazoles, as well as those in terminal nitrogen groups. Transitions involving keto-enol neutral state tautomerism also often lead to sign disagreement.

The current challenge combined log *P* and p*K*_a_ submissions in order to evaluate the current state of log *D* predictions. In general we find that the accuracy of octanol-water log *P* predictions in SAMPL7 is higher than that of cyclohexane-water log *D* predictions in SAMPL5. Half of the methods in the current challenge achieved a RMSE below 2 log *D* units, while no submissions achieved this in the SAMPL5 challenge. Given the abundance of octanol-water partitioning and distribution data (compared to cyclohexane-water data in SAMPL5) it makes sense that accuracy would be higher here in SAMPL7 since trained methods (i.e. empirical methods and implicit solvent QM) are impacted by availability of training data.

## Supplementary Information

Below is the link to the electronic supplementary material.

Supplementary Information 1 (PDF 340 kb)

## Data Availability

All SAMPL7 physical property instructions, submissions, experimental data and analysis are available at https://github.com/samplchallenges/SAMPL7/tree/master/physical_property. Figures and supporting material for this paper can be found at https://github.com/MobleyLab/sampl7-physical-property-challenge-manuscript. This repository contains graphs and plots from the paper, some of which are available in the main SAMPL7 physical property repository listed directly above, but also includes: A graph that shows the distribution of molecular properties of the 22 compounds from the SAMPL7 physical property blind challenge; details of MM-based physical methods that made log *P* predictions; a table that lists additional info for microscopic p*K*_a_ predictions (the table lists the microstate, total number of relative free energy predictions, average relative free energy prediction, average relative free energy prediction, STD, minimum relative free energy prediction, maximum relative free energy prediction, number of (+) sign predictions, number of (-) sign predictions, number of neutral (0) sign predictions, and Shannon entropy (H)); a table of the number of states per charge state for the microstates used in the SAMPL7 p*K*_a_ challenge; a table of the SAMPL7 molecule ID, compound class, and isomeric SMILES of SAMPL7 physical property challenge molecules; structures of the molecules in the SAMPL7 physical property challenge grouped by compound class; a figure showing an example of a relative free energy network; a figure showing chemical transformations that repeatedly show up as having large disagreement on the sign of the relative free energy prediction in the p*K*_a_ challenge; structures of microstates where relative microstate free energy predictions disagree for the p*K*_a_ challenge; a figure showing the Shannon entropy per microstate transition in the p*K*_a_ challenge.

## Abbreviations

SAMPL: Statistical Assessment of the Modeling of Proteins and Ligands
log *P*: log$_{10}$ of the organic solvent-water partition coefficient ($K_{ow}$) of neutral species
log *D*: log$_{10}$ of organic solvent-water distribution coefficient ($D_{ow}$)
p*K*_a_: −log$_{10}$ of the acid dissociation equilibrium constant
SEM: Standard error of the mean
RMSE: Root mean squared error
MAE: Mean absolute error
$\tau$: Kendall’s rank correlation coefficient (Tau)
R^2^: Coefficient of determination (R-Squared)
QM: Quantum mechanics
MM: Molecular mechanics
DL: Database lookup
LFER: Linear free energy relationship
QSPR: Quantitative structure-property relationship
ML: Machine learning
LEC: Linear empirical correction

## References

[1] Manallack DT (2007). The p $${\mathit{K}_{\rm a}}$$ distribution of drugs: application to drug discovery. Perspect Med Chem.

[2] Charifson PS, Walters WP (2014). Acidic and basic drugs in medicinal chemistry: a perspective. J Med Chem.

[3] Aguilar B, Anandakrishnan R, Ruscio JZ, Onufriev AV (2010). Statistics and physical origins of pK and ionization state changes upon protein-ligand binding. Biophys J.

[4] Rupp M, Korner RV, Tetko I (2011). Predicting the pKa of small molecules. CCHTS.

[5] Meanwell NA (2011). Improving drug candidates by design: a focus on physicochemical properties as a means of improving compound disposition and safety. Chem Res Toxicol.

[6] Giaginis C, Tsantili-Kakoulidou A (2008). Alternative measures of lipophilicity: from octanol-water partitioning to IAM retention. J Pharm Sci.

[7] Lang BE (2012). Solubility of water in octan-1-Ol from (275 to 369) K. J Chem Eng Data.

[8] Nicholls A, Mobley DL, Guthrie JP, Chodera JD, Bayly CI, Cooper MD, Pande VS (2008). Predicting small-molecule solvation free energies: an informal blind test for computational chemistry. J Med Chem.

[9] Guthrie JP (2009). A blind challenge for computational solvation free energies: introduction and overview. J Phys Chem B.

[10] Mobley DL, Liu S, Cerutti DS, Swope WC, Rice JE (2012). Alchemical prediction of hydration free energies for SAMPL. J Comput Aided Mol Des.

[11] Geballe MT, Skillman AG, Nicholls A, Guthrie JP, Taylor PJ (2010). The SAMPL2 blind prediction challenge: introduction and overview. J Comput Aided Mol Des.

[12] Mobley DL, Wymer KL, Lim NM, Guthrie JP (2014). Blind prediction of solvation free energies from the SAMPL4 challenge. J Comput Aided Mol Des.

[13] Muddana HS, Fenley AT, Mobley DL, Gilson MK (2014). The SAMPL4 host-guest blind prediction challenge: an overview. J Comput Aided Mol Des.

[14] Yin J, Henriksen NM, Slochower DR, Shirts MR, Chiu MW, Mobley DL, Gilson MK (2017). Overview of the SAMPL5 host-guest challenge: are we doing better?. J Comput Aided Mol Des.

[15] Rizzi A, Jensen T, Slochower DR, Aldeghi M, Gapsys V, Ntekoumes D, Bosisio S, Papadourakis M, Henriksen NM, de Groot BL, Cournia Z, Dickson A, Michel J, Gilson MK, Shirts MR, Mobley DL, Chodera JD (2020). The SAMPL6 SAMPLing challenge: assessing the reliability and efficiency of binding free energy calculations. J Comput Aided Mol Des.

[16] Rizzi A, Murkli S, McNeill JN, Yao W, Sullivan M, Gilson MK, Chiu MW, Isaacs L, Gibb BC, Mobley DL, Chodera JD (2018). Overview of the SAMPL6 host-guest binding affinity prediction challenge. J Comput Aided Mol Des.

[17] Amezcua M, El Khoury L, Mobley DL (2021). SAMPL7 host-guest challenge overview: assessing the reliability of polarizable and non-polarizable methods for binding free energy calculations. J Comput Aided Mol Des.

[18] Muddana HS, Daniel Varnado C, Bielawski CW, Urbach AR, Isaacs L, Geballe MT, Gilson MK (2012). Blind prediction of host-guest binding affinities: a new SAMPL3 challenge. J Comput Aided Mol Des.

[19] Khalak Y, Tresadern G, de Groot BL, Gapsys V (2021). Non-equilibrium approach for binding free energies in cyclodextrins in SAMPL7: force fields and software. J Comput Aided Mol Des.

[20] Mobley DL, Liu S, Lim NM, Wymer KL, Perryman AL, Forli S, Deng N, Su J, Branson K, Olson AJ (2014). Blind prediction of HIV integrase binding from the SAMPL4 challenge. J Comput Aided Mol Des.

[21] Benson ML, Faver JC, Ucisik MN, Dashti DS, Zheng Z, Merz KM (2012). Prediction of trypsin/molecular fragment binding affinities by free energy decomposition and empirical scores. J Comput Aided Mol Des.

[22] Gallicchio E, Deng N, He P, Wickstrom L, Perryman AL, Santiago DN, Forli S, Olson AJ, Levy RM (2014). Virtual screening of integrase inhibitors by large scale binding free energy calculations: the SAMPL4 challenge. J Comput Aided Mol Des.

[23] Hogues H, Sulea T, Purisima EO (2014). Exhaustive docking and solvated interaction energy scoring: lessons learned from the SAMPL4 challenge. J Comput Aided Mol Des.

[24] Kulp JL, Blumenthal SN, Wang Q, Bryan RL, Guarnieri F (2012). A fragment-based approach to the SAMPL3 challenge. J Comput Aided Mol Des.

[25] Kumar A, Zhang KYJ (2012). Computational fragment-based screening using rosettaligand: the SAMPL3 challenge. J Comput Aided Mol Des.

[26] Deng N, Forli S, He P, Perryman A, Wickstrom L, Vijayan RSK, Tiefenbrunn T, Stout D, Gallicchio E, Olson AJ, Levy RM (2015). Distinguishing binders from false positives by free energy calculations: fragment screening against the flap site of HIV protease. J Phys Chem B.

[27] Işık M, Levorse D, Rustenburg AS, Ndukwe IE, Wang H, Wang X, Reibarkh M, Martin GE, Makarov AA, Mobley DL, Rhodes T, Chodera JD (2018). pKa measurements for the SAMPL6 prediction challenge for a set of kinase inhibitor-like fragments. J Comput Aided Mol Des.

[28] Selwa E, Kenney IM, Beckstein O, Iorga BI (2018). SAMPL6: calculation of macroscopic pKa values from Ab initio quantum mechanical free energies. J Comput Aided Mol Des.

[29] Bannan CC, Mobley DL, Skillman AG (2018). SAMPL6 challenge results from *K*_a_ redictions based on a general Gaussian process model. J Comput Aided Mol Des.

[30] Zeng Q, Jones MR, Brooks BR (2018). Absolute and relative pKa predictions via a DFT approach applied to the SAMPL6 blind challenge. J Comput Aided Mol Des.

[31] Tielker N, Eberlein L, Güssregen S, Kast SM (2018). The SAMPL6 challenge on predicting aqueous pKa values from EC-RISM theory. J Comput Aided Mol Des.

[32] Pracht P, Wilcken R, Udvarhelyi A, Rodde S, Grimme S (2018). High accuracy quantum-chemistry-based calculation and blind prediction of macroscopic pKa values in the context of the SAMPL6 challenge. J Comput Aided Mol Des.

[33] Prasad S, Huang J, Zeng Q, Brooks BR (2018). An explicit-solvent hybrid QM and MM approach for predicting pKa of small molecules in SAMPL6 challenge. J Comput Aided Mol Des.

[34] Bannan CC, Burley KH, Chiu M, Shirts MR, Gilson MK, Mobley DL (2016). Blind prediction of cyclohexane-water distribution coefficients from the SAMPL5 challenge. J Comput Aided Mol Des.

[35] Rustenburg AS, Dancer J, Lin B, Feng JA, Ortwine DF, Mobley DL, Chodera JD (2016). Measuring experimental cyclohexane-water distribution coefficients for the SAMPL5 challenge. J Comput Aided Mol Des.

[36] Kamath G, Kurnikov I, Fain B, Leontyev I, Illarionov A, Butin O, Olevanov M, Pereyaslavets L (2016). Prediction of cyclohexane-water distribution coefficient for SAMPL5 drug-like compounds with the QMPFF3 and ARROW polarizable force fields. J Comput Aided Mol Des.

[37] Klamt A, Eckert F, Reinisch J, Wichmann K (2016). Prediction of Cyclohexane-water distribution coefficients with COSMO-RS on the SAMPL5 data set. J Comput Aided Mol Des.

[38] Işık M, Bergazin TD, Fox T, Rizzi A, Chodera JD, Mobley DL (2020). Assessing the accuracy of octanol-water partition coefficient predictions in the SAMPL6 Part II Log P challenge. J Comput Aided Mol Des.

[39] Fan S, Iorga BI, Beckstein O (2020). Prediction of octanol-water partition coefficients for the SAMPL6-log P molecules using molecular dynamics simulations with OPLS-AA, AMBER and CHARMM force fields. J Comput Aided Mol Des.

[40] Zamora WJ, Pinheiro S, German K, Ràfols C, Curutchet C, Luque FJ (2020). Prediction of the N-octanol/water partition coefficients in the SAMPL6 blind challenge from MST continuum solvation calculations. J Comput Aided Mol Des.

[41] Jones MR, Brooks BR (2020). Quantum chemical predictions of water-octanol partition coefficients applied to the SAMPL6 logP blind challenge. J Comput Aided Mol Des.

[42] Pickard FC, König G, Tofoleanu F, Lee J, Simmonett AC, Shao Y, Ponder JW, Brooks BR (2016). Blind prediction of distribution in the SAMPL5 challenge with QM based protomer and pK a corrections. J Comput Aided Mol Des.

[43] Işık M, Rustenburg AS, Rizzi A, Gunner MR, Mobley DL, Chodera JD (2021). Overview of the SAMPL6 pKa challenge: evaluating small molecule microscopic and macroscopic pKa predictions. J Comput Aided Mol Des.

[44] Işık M, Levorse D, Mobley DL, Rhodes T, Chodera JD (2020). Octanol-water partition coefficient measurements for the SAMPL6 blind prediction challenge. J Comput Aided Mol Des.

[45] ACD/pKa Classic (ACD/Percepta Kernel v1.6) (2018) Advanced Chem-istry Development, Inc., Toronto, ON, Canada. https://www.acdlabs.com/products/percepta/predictors/pKa/. Accessed 26 May 2018

[46] Shelley JC, Cholleti A, Frye LL, Greenwood JR, Timlin MR, Uchimaya M (2007). Epik: a software program for pK a prediction and protonation state generation for drug-like molecules. J Comput Aided Mol Des.

[47] MoKa (2018). Molecular discovery.

[48] Simulations Plus ADMET Predictor v8.5 (2018) Simulations Plus, Lancaster, CA. https://www.simulations-plus.com/software/admetpredictor/physicochemical-biopharmaceutical/. Accessed 15 Mar 2021

[49] Tissandier MD, Cowen KA, Feng WY, Gundlach E, Cohen MH, Earhart AD, Coe JV, Tuttle TR (1998). The proton’s absolute aqueous enthalpy and gibbs free energy of solvation from cluster-ion solvation data. J Phys Chem A.

[50] Klamt A, Eckert F, Diedenhofen M, Beck ME (2003). First principles calculations of aqueous p $${{{\mathit{K}}}}_{{\rm a}}$$ values for organic and inorganic acids using COSMO-RS reveal an inconsistency in the slope of the p $${{{\mathit{K}}}}_{{\rm a}}$$ scale. J Phys Chem A.

[51] Alongi KS, Shields GC (2010). Theoretical calculations of acid dissociation constants: a review article. Annu Rep Comput Chem.

[52] Liao C, Nicklaus MC (2009). Comparison of nine programs predicting p $${{{\mathit{K}}}}_{{\rm a}}$$ values of pharmaceutical substances. J Chem Inf Model.

[53] Bochevarov AD, Watson MA, Greenwood JR, Philipp DM (2016). Multiconformation, density functional theory-based p $${{{\mathit{K}}}}_{{\rm a}}$$ prediction in application to large, flexible organic molecules with diverse functional groups. J Chem Theory Comput.

[54] Tielker N, Eberlein L, Chodun C, Güssregen S, Kast SM (2019). pKa calculations for tautomerizable and conformationally flexible molecules: partition function vs. state transition approach. J Chem Theory Comput.

[55] Gunner MR, Murakami T, Rustenburg AS, Işık M, Chodera JD (2020). Standard state free energies, not pKas, are ideal for describing small molecule protonation and tautomeric states. J Comput Aided Mol Des.

[56] Marenich AV, Cramer CJ, Truhlar DG (2009). Universal solvation model based on solute electron density and on a continuum model of the solvent defined by the bulk dielectric constant and atomic surface tensions. J Phys Chem B.

[57] Marenich AV, Cramer CJ, Truhlar DG (2013). Generalized born solvation model SM12. J Chem Theory Comput.

[58] Loschen C, Reinisch J, Klamt A (2019). COSMO-RS based predictions for the SAMPL6 logP challenge. J Comput Aided Mol Des.

[59] Klamt A, Eckert F, Diedenhofen M (2009). Prediction of the free energy of hydration of a challenging set of pesticide-like compounds. J Phys Chem B.

[60] Klamt A (1995). Conductor-like screening model for real solvents: a new approach to the quantitative calculation of solvation phenomena. J Phys Chem.

[61] Klamt A, Jonas V, Bürger T, Lohrenz JCW (1998). Refinement and parametrization of COSMO-RS. J Phys Chem A.

[62] Li H, Chowdhary J, Huang L, He X, MacKerell AD, Roux B (2017). Drude polarizable force field for molecular dynamics simulations of saturated and unsaturated Zwitterionic lipids. J Chem Theory Comput.

[63] Wang J, Wolf RM, Caldwell JW, Kollman PA, Case DA (2004). Development and testing of a general amber force field. J Comput Chem.

[64] Vassetti D, Pagliai M, Procacci P (2019). Assessment of GAFF2 and OPLS-AA general force fields in combination with the water models TIP3P, SPCE, and OPC3 for the solvation free energy of druglike organic molecules. J Chem Theory Comput.

[65] Vanommeslaeghe K, Hatcher E, Acharya C, Kundu S, Zhong S, Shim J, Darian E, Guvench O, Lopes P, Vorobyov I, Mackerell AD (2009). CHARMM general force field: a force field for drug-like molecules compatible with the CHARMM all-atom additive biological force fields. J Comput Chem.

[66] Dodda LS, Cabeza de Vaca I, Tirado-Rives J, Jorgensen WL (2017). LigParGen web server: an automatic OPLS-AA parameter generator for organic ligands. Nucleic Acids Res.

[67] Jorgensen WL, Chandrasekhar J, Madura JD, Impey RW, Klein ML (1983). Comparison of simple potential functions for simulating liquid water. J Chem Phys.

[68] Izadi S, Onufriev AV (2016). Accuracy limit of rigid 3-point water models. J Chem Phys.

[69] Procacci P, Cardelli C (2014). Fast switching alchemical transformations in molecular dynamics simulations. J Chem Theory Comput.

[70] Jarzynski C (1997). Nonequilibrium equality for free energy differences. Phys Rev Lett.

[71] Zwanzig RW (1954). High-temperature equation of state by a perturbation method. I. Nonpolar gases. J Chem Phys.

[72] Kirkwood JG (1935). Statistical mechanics of fluid mixtures. J Chem Phys.

[73] Bennett CH (1976). Efficient estimation of free energy differences from Monte Carlo data. J Comput Phys.

[74] Shirts MR, Chodera JD (2008). Statistically optimal analysis of samples from multiple equilibrium states. J Chem Phys..

[75] Prasad S, Brooks BR (2020). A deep learning approach for the blind logP prediction in SAMPL6 challenge. J Comput Aided Mol Des.

[76] Schroeter TS, Schwaighofer A, Mika S, Ter Laak A, Suelzle D, Ganzer U, Heinrich N, Müller KR (2007). Predicting lipophilicity of drug-discovery molecules using Gaussian Process models. ChemMedChem.

[77] Francisco KR, Varricchio C, Paniak TJ, Kozlowski MC, Brancale A, Ballatore C (2021). Structure property relationships of N-Acylsulfonamides and related bioisosteres. Eur J Med Chem.

[78] RDKit: Open-source cheminformatics. http://www.rdkit.org. Accessed 20 Mar 2021

[79] Quacpac Toolkit 2020.2.0 OpenEye Scientific Software, Santa Fe, NM. http://www.eyesopen.com. Accessed Feb 2020

[80] Chemicalize Toolkit: Property and structure calculator. Developed by ChemAxon. https://chemicalize.com/. Accessed Feb 2020

[81] Greenwood JR, Calkins D, Sullivan AP, Shelley JC (2010). Towards the comprehensive, rapid, and accurate prediction of the favorable tautomeric states of drug-like molecules in aqueous solution. J Comput Aided Mol Des.

[82] Tielker N, Tomazic D, Heil J, Kloss T, Ehrhart S, Güssregen S, Schmidt KF, Kast SM (2016). The SAMPL5 challenge for embedded-cluster integral equation theory: solvation free energies, aqueous pK a, and cyclohexane-water Log D. J Comput Aided Mol Des.

[83] Tielker N, Eberlein L, Hessler G, Schmidt KF, Güssregen S, Kast SM (2021). Quantum-mechanical property prediction of solvated drug molecules: what have we learned from a decade of SAMPL blind prediction challenges?. J Comput Aided Mol Des.

[84] Gao F, Wolf G, Hirn M (2019) Geometric Scattering for Graph Data Analysis. In: *International Conference on Machine Learning* PMLR, pp 2122–2131

[85] Donyapour N, Dickson A (2021). Predicting partition coefficients for the SAMPL7 physical property challenge using the ClassicalGSG method. J Comput Aided Mol Des.

[86] Donyapour N, Hirn M, Dickson A (2021). ClassicalGSG: prediction of Log P using classical molecular force fields and geometric scattering for graphs. J Comput Chem.

[87] Perez KL, Pinheiro S, Zamora W (2021) Multiple linear regression models for predicting the n-Octanol/water partition coefficients in the SAMPL7 blind challenge. J Comput Aided Mol Des 10.1007/s10822-021-00409-2 PMC8273033 34251523

[88] Lenselink EB, Stouten PFW (2021) Multitask machine learning models for predicting lipophilicity (logP). J Comput Aided Mol Des 10.1007/s10822-021-00405-6 PMC8367913 34273053

[89] Warnau J, Wichmann K, Reinisch J (2021) COSMO-RS predictions of LogP in the SAMPL7 blind challenge. J Comput Aided Mol Des 10.1007/s10822-021-00395-5 34125358

[90] Viayna A, Pinheiro S, Curutchet C, Luque FJ, Zamora WJ (2021) Prediction of n-octanol/water partition coefficients and acidity constants (pKa) in the SAMPL7 blind challenge with the IEFPCM-MST model. J Comput Aided Mol Des 10.1007/s10822-021-00394-6 PMC8295120 34244905

[91] Fan S, Nedev H, Vijayan R, Iorga BI, Beckstein O (2021) Precise force-field-based calculations of octanol-water partition coefficients for the SAMPL7 molecules. J Comput Aided Mol Des 10.1007/s10822-021-00407-4 PMC8397498 34232435

[92] Tielker N, Güssregen S, Kast SM (2021) SAMPL7 physical property prediction from EC-RISM theory. J Comput Aided Mol Des 10.1007/s10822-021-00410-9 PMC8367877 34278539

[93] Falcioni F, Kalayan J, Henchman R (2021) Energy-entropy prediction of octanol-water LogP of SAMPL7 N-Acyl sulfonamideBioisoesters. J Comput Aided Mol Des 10.1007/s10822-021-00401-w PMC8295089 34244906

[94] Fındık BK, Haslak ZP, Arslan E, Aviyente V (2021) SAMPL7 blind challenge: quantum-mechanical prediction of partition coefficients and acid dissociation constants for small drug-like molecules. J Comput Aided Mol Des 10.1007/s10822-021-00402-9 34164769

[95] Kim S, Chen J, Cheng T, Gindulyte A, He J, He S, Li Q, Shoemaker BA, Thiessen PA, Yu B, Zaslavsky L, Zhang J, Bolton EE (2021). PubChem in 2021: new data content and improved web interfaces. Nucleic Acids Res.

[96] DrugBank: Online database of drug and drug target information;. https://www.drugbank.com/. Accessed 15 Aug 2020

[97] O’Boyle NM, Banck M, James CA, Morley C, Vandermeersch T, Hutchison GR (2011). Open babel: an open chemical toolbox. J Cheminf.

[98] Chemprop: Directed message passing neural network;. https://chemprop.readthedocs.io/en/latest/

[99] Yang K, Swanson K, Jin W, Coley C, Eiden P, Gao H, Guzman-Perez A, Hopper T, Kelley B, Mathea M, Palmer A, Settels V, Jaakkola T, Jensen K, Barzilay R (2019). Analyzing learned molecular representations for property prediction. J Chem Inf Model.

[100] COSMOquick: COSMO-RS based toolbox;. https://www.3ds.com/products-services/biovia/products/molecular-modeling-simulation/solvation-chemistry/cosmoquick/. Accessed Oct 2020

[101] COSMOconf: A flexible conformer generator for COSMO-RS;. https://www.3ds.com/products-services/biovia/products/molecular-modeling-simulation/solvation-chemistry/cosmoconf/. Accessed Oct 2020

[102] Balasubramani SG, Chen GP, Coriani S, Diedenhofen M, Frank MS, Franzke YJ, Furche F, Grotjahn R, Harding ME, Hättig C, Hellweg A, Helmich-Paris B, Holzer C, Huniar U, Kaupp M, Marefat Khah A, Karbalaei Khani S, Müller T, Mack F, Nguyen BD (2020). TURBOMOLE: modular program suite for Ab initio quantum-chemical and condensed-matter simulations. J Chem Phys.

[103] TURBOMOLE V7.5. University of Karlsruhe and Forschungszentrum Karlsruhe GmbH, 1989-2007, TURBOMOLE GmbH, since (2007) https://www.turbomole.org. Accessed 25 Mar 2021

[104] BIOVIA COSMOtherm: Tool for predictive property calculation of liquids. Version (2020). Dassault Systemes.;. https://www.3ds.com/products-services/biovia/products/molecular-modeling-simulation/solvation-chemistry/cosmotherm/. Accessed Oct 2020

[105] Miteva MA, Guyon F, Tuffery P (2010) Frog2: Efficient 3D conformation ensemble generator for small compounds. Nucleic Acids Res 38(2):W622–W627. 10.1093/nar/gkq325 10.1093/nar/gkq325 PMC2896087 20444874

[106] Frog v2.14: FRee On line druG conformation generation;. https://bioserv.rpbs.univ-paris-diderot.fr/services/Frog2/

[107] Brown TN, Mora-Diez N (2006). Computational determination of aqueous p $${{{\mathit{K}}}}_{{\rm a}}$$ values of protonated benzimidazoles (Part 2). J Phys Chem B.

[108] Zamora WJ, Curutchet C, Campanera JM, Luque FJ (2017). Prediction of pH-dependent hydrophobic profiles of small molecules from Miertus-Scrocco-Tomasi continuum solvation calculations. J Phys Chem B.

[109] Fraczkiewicz R, Triggle DJ, Taylor JB (2007). In silico prediction of ionization. Comprehensive medicinal chemistry II.

