# Supplementary material for: Evaluation of log P, pKa, and log D predictions from the SAMPL7 blind challenge
Source: J Comput Aided Mol Des. 2021 Jun 24;35(7):771–802. doi: 10.1007/s10822-021-00397-3 (PMC8224998; doi:10.1007/s10822-021-00397-3)
Supplement: Supplementary file 1 — Supplementary Information 1 (PDF 340 kb) [file 10822_2021_397_MOESM1_ESM.pdf]

11 Supplementary Information

11.1 Supplementary figures and tables

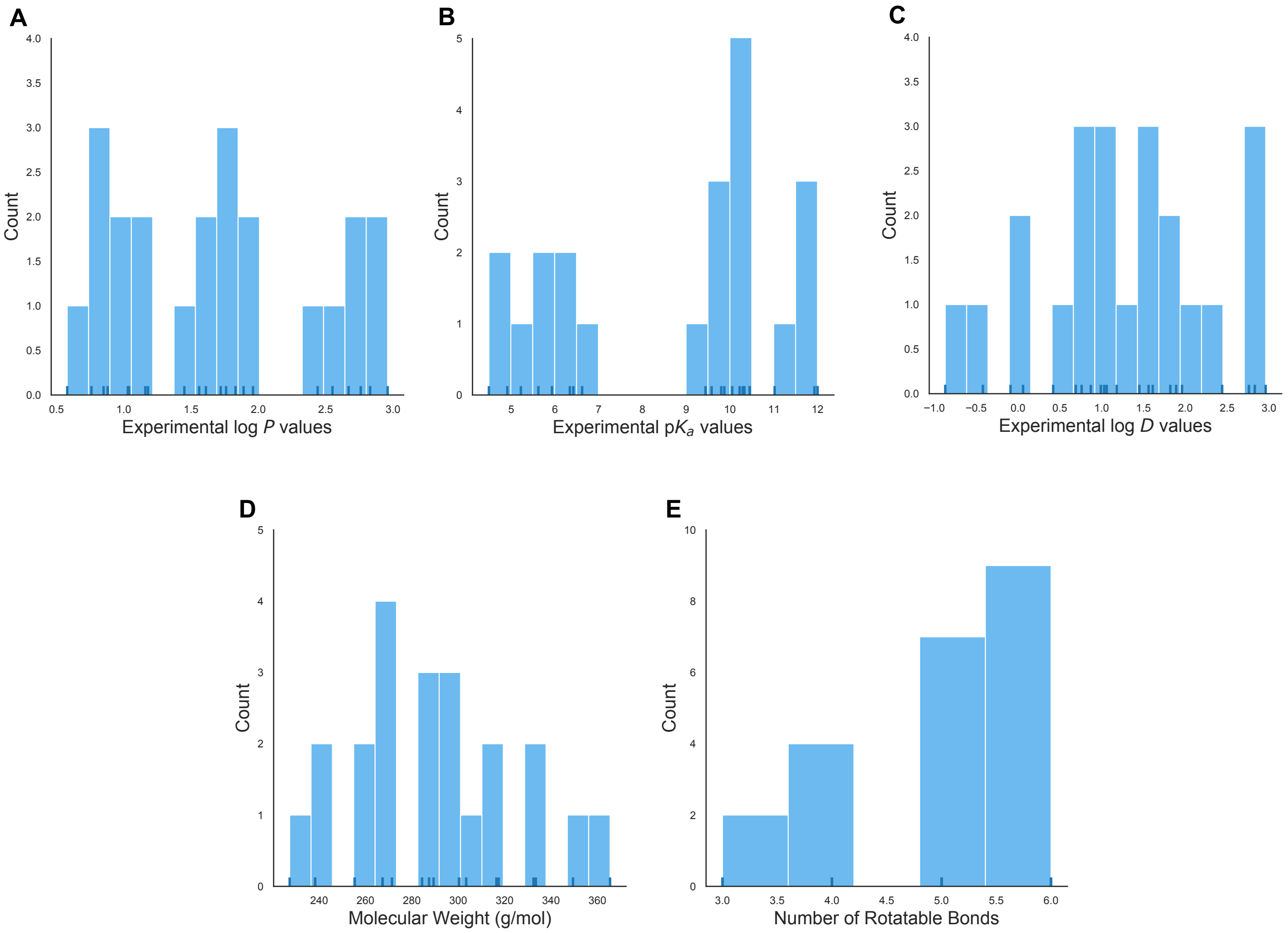

Figure S1. Distribution of molecular properties of the 22 compounds from the SAMPL7 physical property blind challenge. (A) Histogram of log *P* measurements collected with Sirius T3 instrument. The ticks along the x-axis indicate the individual values. Compounds have experimental log *P* values in the range of 0.58-2.96. **(B)** Histogram of pK_a measurements collected with Sirius T3 instrument.. Eight compounds have measured pK_a's in the range of 4.49–6.62 and eleven in the range 9.58- 11.93. Two compounds are included here as having pK_a's of 12, but actually had experimental values greater than 12, and were therefore outside of the experimental detection range. **(C)** Histogram of log *D* measurements between n-octanol and aqueous buffer at pH 7.4 were determined via potentiometric titrations using a Sirius T3 instrument, except for compounds SM27, SM28, SM30-SM34, SM36-SM39 which had log $D_{7.4}$ values determined via shake-flask assay. log *D* measurements ranged from -0.87-2.96. **(D)** Histogram of molecular weights calculated for the compounds in the SAMPL7 dataset. The molecular weight ranged from 227-365 Da. **(E)** Histogram of the number of rotatable bonds in each molecule. The number of rotatable bonds in challenge molecules ranged from 3-6.

Table S1. Evaluation statistics calculated for all methods in the log *P* challenge. Submitted predictions are represented by their method name. There are six error metrics reported: the root-mean-squared error (RMSE), mean absolute error (MAE), mean (signed) error (ME), coefficient of determination (R^2), linear regression slope (m), and Kendall's Rank Correlation Coefficient (τ), and error slope (ES). The mean and 95% confidence intervals of each statistic is presented. This table is ranked by increasing RMSE.

Method Name	Category	Submission Type	RMSE	MAE	ME	R^2	m	Kendall's Tau	ES
ClassicalGSG DB2	Empirical	Blind	0.55 [0.38, 0.69]	0.44 [0.31, 0.58]	0.05 [-0.20, 0.26]	0.51 [0.18, 0.82]	0.71 [0.36, 1.06]	0.51 [0.18, 0.78]	0.81 [0.62, 1.03]
TFE MLR	Empirical	Blind	0.58 [0.34, 0.83]	0.41 [0.26, 0.60]	-0.04 [-0.30, 0.19]	0.43 [0.06, 0.80]	0.60 [0.21, 0.95]	0.56 [0.23, 0.82]	1.38 [1.27, 1.45]
ClassicalGSG DB4	Empirical	Blind	0.65 [0.50, 0.78]	0.55 [0.41, 0.69]	0.25 [0.01, 0.50]	0.51 [0.19, 0.76]	0.82 [0.39, 1.22]	0.45 [0.15, 0.71]	0.57 [0.46, 0.85]
Chemprop	Empirical	Blind	0.66 [0.39, 0.88]	0.48 [0.30, 0.68]	-0.17 [-0.44, 0.08]	0.41 [0.11, 0.76]	0.69 [0.31, 1.08]	0.54 [0.25, 0.82]	1.03 [0.79, 1.21]
TFE-SM8-vacuum-opt	Physical (QM)	Blind	0.67 [0.45, 0.86]	0.51 [0.33, 0.69]	0.15 [-0.13, 0.42]	0.45 [0.11, 0.75]	0.80 [0.33, 1.23]	0.50 [0.18, 0.76]	0.99 [0.75, 1.20]
GROVER	Empirical	Blind	0.69 [0.41, 0.96]	0.49 [0.31, 0.71]	-0.21 [-0.50, 0.05]	0.33 [0.04, 0.70]	0.56 [0.18, 0.92]	0.37 [0.05, 0.66]	0.87 [0.62, 1.09]
ClassicalGSG DB1	Empirical	Blind	0.76 [0.56, 0.96]	0.62 [0.45, 0.82]	0.10 [-0.23, 0.40]	0.28 [0.06, 0.60]	0.61 [0.26, 0.99]	0.36 [0.04, 0.63]	0.63 [0.43, 0.85]
ffsampled_deeplearning_cl1	Empirical	Blind	0.77 [0.44, 1.04]	0.51 [0.29, 0.77]	-0.25 [-0.58, 0.04]	0.31 [0.05, 0.70]	0.63 [0.24, 1.05]	0.42 [0.06, 0.74]	0.99 [0.72, 1.19]
ClassicalGSG DB3	Empirical	Blind	0.77 [0.57, 0.96]	0.62 [0.43, 0.82]	-0.15 [-0.46, 0.16]	0.51 [0.18, 0.78]	1.08 [0.55, 1.59]	0.48 [0.15, 0.75]	0.60 [0.42, 0.89]
COSMO-RS	Physical (QM)	Blind	0.78 [0.49, 1.01]	0.57 [0.36, 0.80]	-0.30 [-0.61, -0.01]	0.49 [0.17, 0.79]	0.97 [0.49, 1.45]	0.53 [0.25, 0.78]	0.97 [0.74, 1.18]
TFE_Attentive_FP	Empirical	Blind	0.79 [0.47, 1.07]	0.57 [0.36, 0.82]	-0.18 [-0.53, 0.12]	0.19 [0.00, 0.61]	0.44 [0.04, 0.87]	0.34 [-0.02, 0.69]	0.93 [0.69, 1.13]
ffsampled_deeplearning_cl2	Empirical	Blind	0.82 [0.48, 1.11]	0.56 [0.32, 0.83]	-0.37 [-0.69, -0.08]	0.36 [0.07, 0.72]	0.73 [0.31, 1.16]	0.40 [0.07, 0.69]	0.94 [0.67, 1.15]
TFE-SM12-vacuum-opt	Physical (QM)	Blind	0.82 [0.61, 1.02]	0.66 [0.47, 0.87]	0.28 [-0.06, 0.60]	0.41 [0.08, 0.72]	0.90 [0.36, 1.42]	0.39 [0.05, 0.67]	0.88 [0.65, 1.09]
TFE-SM8-solvent-opt	Physical (QM)	Blind	0.97 [0.71, 1.20]	0.78 [0.55, 1.02]	0.65 [0.35, 0.94]	0.42 [0.10, 0.70]	0.83 [0.35, 1.31]	0.44 [0.13, 0.69]	0.71 [0.47, 0.94]
REF1 ChemAxon	Empirical	Reference	1.00 [0.79, 1.20]	0.85 [0.63, 1.08]	0.46 [0.08, 0.83]	0.39 [0.10, 0.70]	0.98 [0.45, 1.53]	0.40 [0.09, 0.68]	0.13 [-0.00, 0.29]
TFE IEFPCM MST	Physical (QM)	Blind	1.03 [0.65, 1.41]	0.80 [0.56, 1.10]	-0.07 [-0.53, 0.33]	0.27 [0.01, 0.68]	0.85 [0.12, 1.50]	0.42 [0.10, 0.70]	1.07 [0.88, 1.23]
TFE MD neat oct (GAFF/TIP4P)	Physical (MM)	Blind	1.11 [0.74, 1.43]	0.83 [0.52, 1.15]	-0.74 [-1.10, -0.40]	0.56 [0.24, 0.82]	1.25 [0.64, 1.83]	0.58 [0.27, 0.82]	1.30 [1.19, 1.40]
NULL0 mean clogP FDA	Empirical	Reference	1.20 [0.94, 1.42]	1.01 [0.73, 1.28]	-0.96 [-1.26, -0.64]	0.00 [0.00, 0.00]	0.00 [-0.00, 0.00]	nan [nan, nan]	0.18 [0.04, 0.32]
NES-1 (GAFF2/OPC3) G	Physical (MM)	Blind	1.21 [0.92, 1.51]	1.03 [0.78, 1.31]	-0.13 [-0.63, 0.37]	0.22 [0.01, 0.59]	0.88 [0.15, 1.59]	0.34 [0.02, 0.63]	1.23 [1.11, 1.33]
NES-1 (GAFF2/OPC3) J	Physical (MM)	Blind	1.28 [0.97, 1.58]	1.08 [0.81, 1.38]	0.01 [-0.54, 0.53]	0.21 [0.01, 0.63]	0.92 [0.09, 1.76]	0.33 [0.00, 0.64]	1.21 [1.08, 1.33]
NES-1 (GAFF2/OPC3) B	Physical (MM)	Blind	1.42 [1.02, 1.81]	1.13 [0.79, 1.51]	-0.51 [-1.08, 0.05]	0.27 [0.02, 0.65]	1.11 [0.30, 1.91]	0.36 [0.05, 0.65]	1.17 [1.01, 1.31]
MD (GAFF/TIP3P)	Physical (MM)	Blind	1.43 [1.15, 1.71]	1.30 [1.06, 1.56]	-1.30 [-1.56, -1.06]	0.48 [0.22, 0.79]	0.77 [0.45, 1.12]	0.55 [0.28, 0.80]	0.94 [0.80, 1.09]
TFE wet oct (GAFF/TIP4P)	Physical (MM)	Blind	1.47 [1.16, 1.77]	1.30 [1.03, 1.60]	-1.30 [-1.60, -1.03]	0.42 [0.10, 0.75]	0.80 [0.30, 1.30]	0.47 [0.14, 0.75]	1.15 [1.03, 1.27]
TFE-NHLBI-TZVP-QM	Physical (QM)	Blind	1.55 [1.19, 1.88]	1.34 [1.02, 1.67]	1.32 [1.00, 1.67]	0.52 [0.19, 0.78]	1.16 [0.59, 1.65]	0.51 [0.19, 0.78]	0.05 [-0.00, 0.17]
0.05 *MD (CGenFF/TIP3P)*	Physical (MM)	Blind	1.63 [1.25, 1.98]	1.41 [1.08, 1.76]	-1.38 [-1.74, -1.02]	0.54 [0.26, 0.82]	1.26 [0.81, 1.76]	0.52 [0.26, 0.76]	0.90 [0.70, 1.07]
EC_RISM_wet	Physical (QM)	Blind	1.84 [1.31, 2.36]	1.49 [1.07, 1.96]	-1.49 [-1.96, -1.06]	0.29 [0.05, 0.68]	0.96 [0.37, 1.57]	0.38 [0.08, 0.67]	0.67 [0.45, 0.90]
TFE-SMD-vacuum-opt	Physical (QM)	Blind	1.96 [1.60, 2.30]	1.76 [1.42, 2.13]	1.76 [1.42, 2.13]	0.44 [0.12, 0.68]	1.04 [0.46, 1.59]	0.41 [0.03, 0.70]	0.68 [0.50, 0.87]
MD-EE-MCC (GAFF-TIP4P-Ew)	Physical (MM)	Blind	2.06 [1.48, 2.59]	1.61 [1.09, 2.17]	-0.93 [-1.70, -0.17]	0.03 [0.00, 0.28]	0.47 [-0.53, 1.49]	0.11 [-0.16, 0.38]	0.76 [0.51, 1.03]
MD (OPLS-AA/TIP4P)	Physical (MM)	Blind	2.19 [1.69, 2.65]	1.82 [1.31, 2.34]	-1.35 [-2.03, -0.60]	0.28 [0.06, 0.58]	1.47 [0.58, 2.55]	0.36 [0.07, 0.62]	0.73 [0.48, 0.97]
TFE b3lypd3	Physical (QM)	Blind	2.19 [1.76, 2.57]	1.98 [1.59, 2.37]	1.98 [1.59, 2.37]	0.40 [0.10, 0.67]	1.06 [0.47, 1.64]	0.45 [0.11, 0.72]	0.22 [0.09, 0.41]
MD LigParGen (OPLS-AA/TIP4P)	Physical (MM)	Blind	2.28 [1.80, 2.71]	1.95 [1.46, 2.44]	0.35 [-0.60, 1.29]	0.07 [0.00, 0.37]	0.83 [-0.51, 2.26]	0.19 [-0.14, 0.50]	0.65 [0.42, 0.88]
TFE-SMD-solvent-opt	Physical (QM)	Blind	2.39 [1.97, 2.78]	2.19 [1.79, 2.60]	2.19 [1.79, 2.60]	0.40 [0.09, 0.67]	1.09 [0.45, 1.67]	0.42 [0.09, 0.68]	0.51 [0.34, 0.68]
Ensemble EPI physprop	Empirical	Blind	2.73 [2.27, 3.16]	2.54 [2.13, 2.98]	2.54 [2.13, 2.98]	0.33 [0.04, 0.64]	-0.30 [-0.49, -0.10]	-0.35 [-0.60, -0.03]	-0.00 [-0.00, -0.00]
Ensemble Martel	Empirical	Blind	3.29 [2.89, 3.68]	3.16 [2.78, 3.55]	3.16 [2.78, 3.55]	0.39 [0.05, 0.73]	-0.25 [-0.40, -0.09]	-0.46 [-0.72, -0.14]	-0.00 [-0.00, -0.00]
QSPR_Mordred2D_TPOT_AutoML	Empirical	Blind	3.64 [3.01, 4.24]	3.36 [2.80, 3.96]	3.36 [2.80, 3.96]	0.39 [0.10, 0.71]	-0.72 [-1.12, -0.33]	-0.37 [-0.65, -0.04]	-0.00 [-0.00, -0.00]
TFE-NHLBI-NN-IN	Empirical	Blind	3.97 [3.57, 4.34]	3.85 [3.45, 4.25]	3.85 [3.45, 4.25]	0.00 [0.00, 0.15]	0.02 [-0.30, 0.34]	0.02 [-0.23, 0.27]	0.01 [-0.00, 0.02]

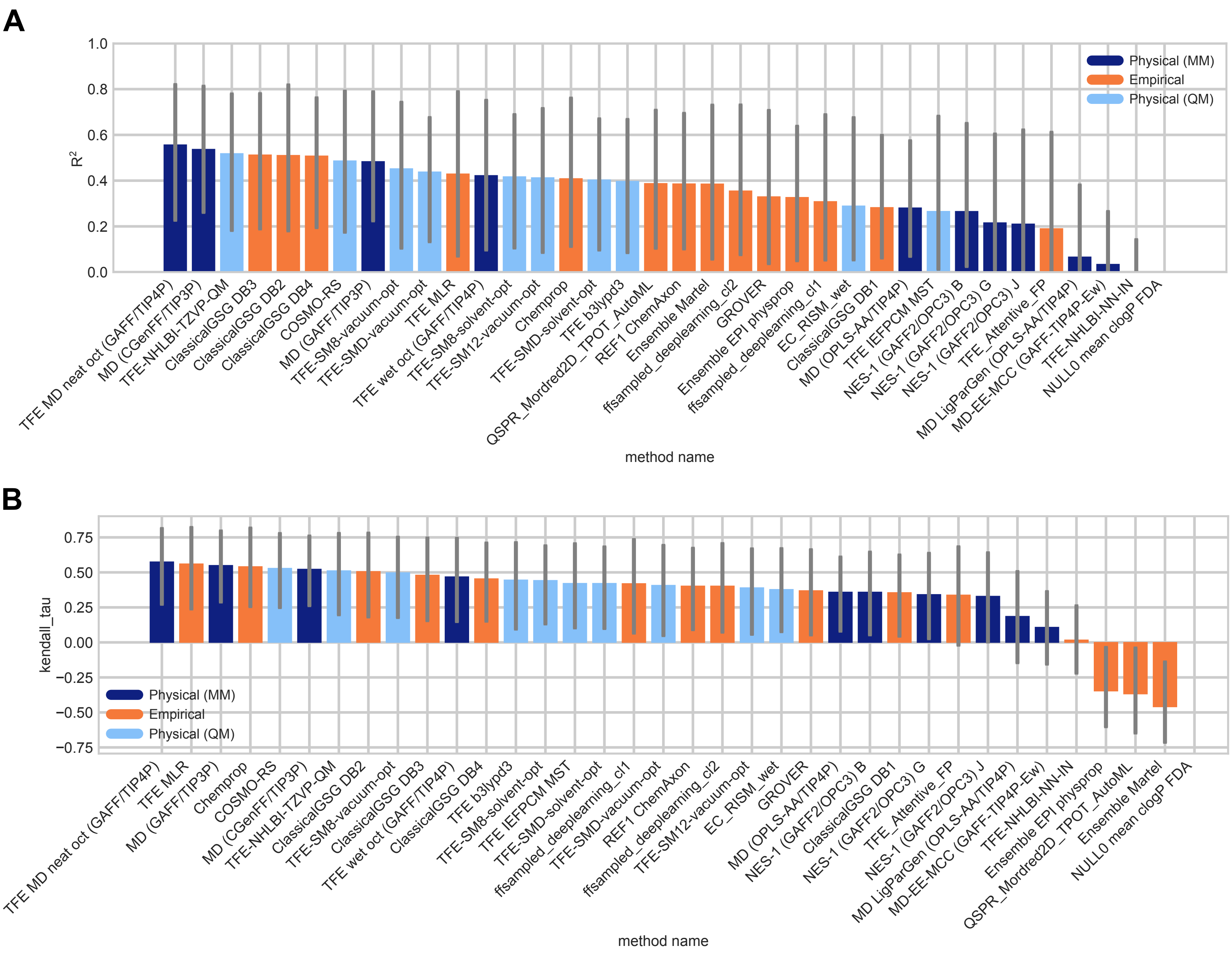

Figure S2. Overall correlation assessment for all methods participating in the SAMPL7 log *P* challenge show that the uncertainty of each correlation statistic is quite high, not allowing a true ranking based on correlation. Pearson's R^2 and Kendall's Rank Correlation Coefficient Tau (τ) are shown, with error bars denoting 95% confidence intervals obtained by bootstrapping over challenge molecules. Submitted methods are listed in Table 1. The submission *REF1 ChemAxon* was a reference method included after the blind challenge submission deadline, and *NULL0 mean cLogP FDA* is the null prediction method; all others refer to blind predictions. Most methods have a statistically indistinguishable performance on ranking because of the small dynamic range of the dataset. Evaluation statistics calculated for all methods are available in Table S1 of the Supplementary Information.

Table S2. Details of MM-based physical methods in the log *P* prediction challenge. Force fields, water models, and octanol phase choice are reported. A dry octanol phase indicates the octanol phase was composed of only octanol. A wet octanol phase indicates the octanol phase was treated as a mixture of octanol and water. RMSE, MAE, R^2, and Kendall's Tau values are reported as mean and 95% confidence intervals.

**Method Name**	**Force Field**	**Water Model**	**Octanol Phase**	**RMSE**	**MAE**	**R^2**	**Kendall's Tau**
TFE MD neat oct (GAFF/TIP4P)	GAFF	TIP4P	dry	1.11 [0.74, 1.43]	0.83 [0.52, 1.15]	0.56 [0.24, 0.82]	0.58 [0.27, 0.82]
NES-1 (GAFF2/OPC3) G	GAFF2	OPC3	dry	1.21 [0.92, 1.51]	1.03 [0.78, 1.31]	0.22 [0.01, 0.59]	0.34 [0.02, 0.63]
NES-1 (GAFF2/OPC3) J	GAFF2	OPC3	dry	1.28 [0.97, 1.58]	1.08 [0.81, 1.38]	0.21 [0.01, 0.63]	0.33 [0.00, 0.64]
NES-1 (GAFF2/OPC3) B	GAFF2	OPC3	dry	1.42 [1.02, 1.81]	1.13 [0.79, 1.51]	0.27 [0.02, 0.65]	0.36 [0.05, 0.65]
MD (GAFF/TIP3P)	GAFF	TIP3P	dry	1.43 [1.15, 1.71]	1.30 [1.06, 1.56]	0.48 [0.22, 0.79]	0.55 [0.28, 0.80]
TFE wet oct (GAFF/TIP4P)	GAFF	TIP4P	wet	1.47 [1.16, 1.77]	1.30 [1.03, 1.60]	0.42 [0.10, 0.75]	0.47 [0.14, 0.75]
MD (CGenFF/TIP3P)	CGenFF	TIP3P	dry	1.63 [1.25, 1.98]	1.41 [1.08, 1.76]	0.54 [0.26, 0.82]	0.52 [0.26, 0.76]
MD-EE-MCC (GAFF-TIP4P-Ew)	GAFF	TIP4P-eW	dry	2.06 [1.48, 2.59]	1.61 [1.09, 2.17]	0.03 [0.00, 0.28]	0.11 [-0.16, 0.38]
MD (OPLS-AA/TIP4P)	OPLS-AA	TIP4P	dry	2.19 [1.69, 2.65]	1.82 [1.31, 2.34]	0.28 [0.06, 0.58]	0.36 [0.07, 0.62]
MD LigParGen (OPLS-AA/TIP4P)	OPLS-AA	TIP4P	dry	2.28 [1.80, 2.71]	1.95 [1.46, 2.44]	0.07 [0.00, 0.37]	0.19 [-0.14, 0.50]

Table S3. Evaluation statistics calculated for all methods in the pK_a challenge. Submitted predictions are represented by their method name. There are six error metrics reported: the root-mean-squared error (RMSE), mean absolute error (MAE), mean (signed) error (ME), coefficient of determination (R^2), linear regression slope (m), Kendall's Rank Correlation Coefficient (τ), and error slope (ES). The mean and 95% confidence intervals of each statistic is presented. This table is ranked by increasing RMSE.

**Method Name**	**Category**	**Submission Type**	**RMSE**	**MAE**	**ME**	**R^2**	**m**	**Kendall's Tau**	**ES**
REF00_Chemaxon_Chemicalize	QSPR/ML	Reference	0.71 [0.50, 0.90]	0.56 [0.38, 0.76]	0.09 [-0.23, 0.38]	0.91 [0.86, 0.96]	0.88 [0.72, 1.02]	0.73 [0.51, 0.90]	0.83 [0.58, 1.04]
EC_RISM	QM	Blind	0.72 [0.45, 0.95]	0.53 [0.33, 0.75]	0.20 [-0.10, 0.50]	0.93 [0.87, 0.98]	0.80 [0.72, 0.91]	0.81 [0.63, 0.96]	1.32 [1.19, 1.42]
IEFPCM/MST	QM	Blind	1.82 [1.00, 2.69]	1.30 [0.84, 1.92]	0.25 [-0.46, 1.09]	0.56 [0.22, 0.87]	0.86 [0.53, 1.18]	0.52 [0.22, 0.76]	1.00 [0.80, 1.17]
DFT_M05-2X_SMD	QM	Blind	2.90 [2.04, 3.69]	2.28 [1.53, 3.10]	-0.78 [-2.02, 0.41]	0.03 [0.00, 0.37]	0.15 [-0.32, 0.53]	0.17 [-0.22, 0.54]	0.55 [0.31, 0.81]
TZVP-QM	QM	Blind	2.90 [2.52, 3.25]	2.75 [2.34, 3.14]	1.20 [0.02, 2.33]	0.23 [0.03, 0.60]	-0.11 [-0.20, -0.04]	-0.14 [-0.49, 0.23]	-0.00 [-0.00, -0.00]
Standard Gaussian Process	QSPR/ML	Blind	3.49 [2.76, 4.12]	2.91 [2.06, 3.75]	2.47 [1.38, 3.55]	0.30 [0.10, 0.69]	-0.05 [-0.09, -0.02]	-0.42 [-0.70, -0.08]	1.11 [0.96, 1.24]
DFT_M06-2X_SMD_implicit	QM	Blind	4.16 [2.00, 6.38]	2.80 [1.76, 4.33]	-0.07 [-1.61, 1.95]	0.52 [0.39, 0.78]	1.70 [0.80, 2.77]	0.70 [0.48, 0.88]	0.50 [0.30, 0.70]
DFT_M06-2X_SMD_implicit_SAS	QM	Blind	4.16 [2.03, 6.44]	2.81 [1.80, 4.36]	-0.20 [-1.71, 1.85]	0.50 [0.36, 0.77]	1.64 [0.72, 2.72]	0.56 [0.28, 0.81]	0.14 [0.02, 0.31]
DFT_M06-2X_SMD_explicit_water	QM	Blind	5.12 [1.19, 7.92]	2.56 [0.96, 4.76]	-0.35 [-2.62, 1.93]	0.20 [0.00, 0.81]	1.10 [-0.39, 2.50]	0.46 [0.06, 0.78]	0.52 [0.29, 0.77]
Gaussian_corrected	QM+LEC	Blind	5.36 [4.70, 5.95]	5.12 [4.42, 5.79]	5.12 [4.42, 5.79]	0.76 [0.63, 0.88]	0.35 [0.27, 0.45]	0.60 [0.42, 0.76]	0.00 [-0.00, 0.00]

Table S4. Evaluation statistics calculated for all log *D* estimates. Predictions are represented a name based on method names participants submitted to the and log *P* challenges. There are six error metrics reported: the root-mean-squared error (RMSE), mean absolute error (MAE), mean (signed) error (ME), coefficient of determination (R^2), linear regression slope (m), Kendall's Rank Correlation Coefficient (τ), and error slope (ES). The mean and 95% confidence intervals of each statistic is presented. This table is ranked by increasing RMSE.

Method Name	Category	Submission Type	RMSE	MAE	ME	R^2	m	Kendall's Tau	ES
REF0 ChemAxon	Empirical	Reference	1.06 [0.82, 1.27]	0.91 [0.68, 1.14]	0.28 [-0.14, 0.70]	0.27 [0.01, 0.58]	0.54 [0.10, 0.90]	0.31 [-0.02, 0.61]	0.12 [-0.00, 0.28]
TFE IEFPCM MST + IEFPCM/MST	Physical (QM)	Standard	1.27 [0.85, 1.64]	0.98 [0.67, 1.33]	0.24 [-0.28, 0.75]	0.55 [0.17, 0.87]	1.31 [0.71, 1.70]	0.57 [0.27, 0.82]	1.16 [0.89, 1.25]
NULL0	Empirical	Reference	1.59 [1.22, 1.93]	1.35 [1.00, 1.71]	1.23 [0.81, 1.65]	0.00 [0.00, 0.00]	0.00 [0.00, 0.00]	nan [nan, nan]	0.65 [0.44, 0.87]
EC_RISM	Physical (QM)	Standard	1.69 [1.30, 2.05]	1.43 [1.07, 1.82]	-1.43 [-1.81, -1.07]	0.53 [0.20, 0.77]	0.95 [0.54, 1.29]	0.51 [0.21, 0.74]	0.84 [0.64, 1.02]
TFE-NHLBI-TZVP-QM + TZVP-QM	Physical (QM)	Standard	1.72 [1.30, 2.12]	1.47 [1.12, 1.86]	1.26 [0.78, 1.75]	0.25 [0.01, 0.64]	0.64 [0.08, 1.25]	0.38 [0.02, 0.70]	0.05 [-0.00, 0.18]
TFE b3lypd3 + DFT_M05-2X_SMD	Physical (QM)	Standard	2.15 [1.56, 2.71]	1.78 [1.31, 2.31]	1.78 [1.31, 2.31]	0.32 [0.04, 0.66]	0.80 [0.27, 1.30]	0.41 [0.05, 0.72]	0.42 [0.27, 0.70]
MD (CGenFF/TIP3P) + Gaussian_corrected	Physical (MM) + QM+LEC	Standard	2.27 [1.97, 2.55]	2.13 [1.80, 2.45]	1.84 [1.21, 2.35]	0.62 [0.35, 0.84]	1.53 [0.93, 2.18]	0.62 [0.36, 0.82]	0.88 [0.75, 1.00]
TFE-SMD-solvent-opt + DFT_M06-2X_SMD_explicit_water	Physical (QM)	Standard	4.54 [2.09, 7.15]	2.92 [1.88, 4.57]	2.88 [1.80, 4.55]	0.25 [0.11, 0.76]	1.92 [0.53, 4.45]	0.55 [0.22, 0.80]	0.55 [0.38, 0.73]

Table S5. Additional info for microscopic pK_a predictions.

Microstate	Total number of relative free energy predictions	Average relative free energy prediction	Average relative free energy prediction STD	Minimum relative free energy prediction	Maximum relative free energy prediction	Number of (+) sign predictions	Number of (-) sign predictions	Number of neutral (0) sign predictions	Shannon entropy (H)
SM25_micro001	9	-0.6	13.2	-15.6	16.3	4	5	0	0.7
SM25_micro002	8	8.8	10.6	-7.5	20.4	6	2	0	0.6
SM25_micro003	8	9.6	2.7	4.5	12.6	8	0	0	0.0
SM25_micro004	2	-8.9	4.5	-12.1	-5.8	0	2	0	0.0
SM25_micro005	2	-0.8	2.1	-2.3	0.7	1	1	0	0.7
SM26_micro001	9	7.3	2.4	3.0	10.7	9	0	0	0.0
SM26_micro002	8	-6.7	20.5	-31.7	22.1	3	5	0	0.7
SM26_micro003	8	20.9	12.0	0.9	32.4	8	0	0	0.0
SM26_micro004	2	4.3	0.7	3.8	4.8	2	0	0	0.0
SM26_micro005	2	8.1	2.6	6.3	10.0	2	0	0	0.0
SM27_micro001	9	13.4	4.9	6.1	19.0	9	0	0	0.0
SM28_micro001	9	-5.7	25.0	-39.0	23.5	4	5	0	0.7
SM28_micro002	8	17.1	8.0	8.2	26.5	8	0	0	0.0
SM28_micro003	8	0.9	8.3	-10.0	12.6	4	4	0	0.7
SM28_micro004	2	25.1	9.1	18.7	31.5	2	0	0	0.0
SM29_micro001	9	12.6	4.3	6.3	18.7	9	0	0	0.0
SM30_micro001	9	12.3	4.2	5.9	17.7	9	0	0	0.0
SM31_micro001	9	13.2	4.4	6.0	18.1	9	0	0	0.0
SM31_micro002	3	-0.6	6.6	-8.1	4.5	2	1	0	0.6
SM32_micro001	9	12.8	4.6	5.9	18.9	9	0	0	0.0
SM33_micro001	9	11.9	3.9	5.2	17.1	9	0	0	0.0
SM34_micro001	9	13.0	4.6	5.7	19.7	9	0	0	0.0
SM34_micro002	3	-0.9	6.4	-8.1	4.4	2	1	0	0.6
SM35_micro001	9	11.7	4.5	3.2	16.2	9	0	0	0.0
SM35_micro002	8	0.2	1.4	-1.9	2.5	5	2	1	0.9
SM35_micro003	8	12.2	5.6	3.2	18.1	8	0	0	0.0
SM36_micro001	9	10.8	3.1	5.2	14.9	9	0	0	0.0
SM36_micro002	8	1.2	1.8	0.0	4.4	4	1	3	1.0
SM36_micro003	8	10.7	3.3	5.2	14.7	8	0	0	0.0
SM37_micro001	9	0.1	9.4	-11.7	13.7	5	4	0	0.7
SM37_micro002	8	9.8	2.9	3.7	12.7	8	0	0	0.0
SM37_micro003	8	0.7	1.8	-1.5	4.2	4	3	1	1.0
SM37_micro004	8	8.9	3.0	3.8	12.4	8	0	0	0.0
SM37_micro005	7	-2.7	7.6	-10.6	11.0	3	4	0	0.7
SM38_micro001	9	11.6	4.6	5.2	17.5	9	0	0	0.0
SM39_micro001	9	10.1	3.1	5.1	14.6	9	0	0	0.0
SM40_micro001	9	10.8	3.3	5.0	15.7	9	0	0	0.0
SM40_micro002	8	-1.8	10.3	-15.5	11.8	4	4	0	0.7
SM41_micro001	9	8.4	3.5	2.2	14.8	9	0	0	0.0
SM41_micro002	8	-0.5	9.9	-12.9	13.9	4	4	0	0.7
SM42_micro001	9	5.5	4.6	0.2	12.3	9	0	0	0.0
SM42_micro002	8	-0.2	8.6	-10.8	14.3	4	4	0	0.7
SM42_micro003	3	-2.0	3.0	-5.1	1.0	1	2	0	0.6
SM43_micro001	9	5.9	4.4	0.5	13.4	9	0	0	0.0
SM43_micro002	8	0.1	9.4	-11.0	11.0	4	4	0	0.7
SM43_micro003	8	-11.6	38.1	-60.9	38.2	4	4	0	0.7
SM43_micro004	2	-3.6	2.2	-5.2	-2.1	0	2	0	0.0
SM43_micro005	2	0.1	0.4	-0.2	0.4	1	1	0	0.7
SM44_micro001	9	9.5	2.9	4.3	12.9	9	0	0	0.0
SM44_micro002	8	-1.1	7.4	-10.3	9.9	4	4	0	0.7
SM45_micro001	9	9.6	3.1	4.4	14.7	9	0	0	0.0
SM45_micro002	8	-1.0	7.8	-11.0	9.6	4	4	0	0.7
SM46_micro001	9	9.9	4.1	4.0	18.4	9	0	0	0.0
SM46_micro002	8	-0.7	7.5	-9.6	10.5	4	4	0	0.7
SM46_micro003	8	-12.2	37.1	-63.5	39.0	4	4	0	0.7
SM46_micro004	3	6.3	4.5	2.4	11.3	3	0	0	0.0

Table S6. SMILES and compound class of SAMPL7 physical property challenge molecules. A view of the compounds and their classes can be found in Figure S3.

SAMPL7 Molecule ID	Compound Class	Isomeric SMILES
SM25	acylsulfonamide	O=C(NS(C1=CC=CC=C1)(=O)=O)CCC2=CC=CC=C2
SM26	acylsulfonamide	O=S(CCC1=CC=CC=C1)(NC(C)=O)=O
SM27	oxetane	O=S(CCC1=CC=CC=C1)(NC2(C)COC2)=O
SM28	thietane-1,1-dioxide	O=S(CC1(NC(C)=O)CCC2=CC=CC=C2)(C1)=O
SM29	oxetane	CS(NC1(COC1)CCC2=CC=CC=C2)(=O)=O
SM30	oxetane	O=S(NC1(COC1)CCC2=CC=CC=C2)(C3=CC=CC=C3)=O
SM31	oxetane	O=S(NC1(COC1)CCC2=CC=CC=C2)(N(C)C)=O
SM32	thietane	CS(NC1(CSC1)CCC2=CC=CC=C2)(=O)=O
SM33	thietane	O=S(NC1(CSC1)CCC2=CC=CC=C2)(C3=CC=CC=C3)=O
SM34	thietane	O=S(NC1(CSC1)CCC2=CC=CC=C2)(N(C)C)=O
SM35	thietane-1-oxide	CS(N[C@@]1(C[S+]([O-])C1)CCC2=CC=CC=C2)(=O)=O
SM36	thietane-1-oxide	O=S(N[C@@]1(C[S+]([O-])C1)CCC2=CC=CC=C2)(C3=CC=CC=C3)=O
SM37	thietane-1-oxide	O=S(N[C@@]1(C[S+]([O-])C1)CCC2=CC=CC=C2)(N(C)C)=O
SM38	thietane-1,1-dioxide	CS(NC1(CS(C1)(=O)=O)CCC2=CC=CC=C2)(=O)=O
SM39	thietane-1,1-dioxide	O=S(NC1(CS(C1)(=O)=O)CCC2=CC=CC=C2)(C3=CC=CC=C3)=O
SM40	thietane-1,1-dioxide	O=S(NC1(CS(C1)(=O)=O)CCC2=CC=CC=C2)(N(C)C)=O
SM41	isoxazole	O=S(NC1=NOC(C2=CC=CC=C2)=C1)(C)=O
SM42	isoxazole	O=S(NC1=NOC(C2=CC=CC=C2)=C1)(C3=CC=CC=C3)=O
SM43	isoxazole	O=S(NC1=NOC(C2=CC=CC=C2)=C1)(N(C)C)=O
SM44	1,2,3-triazole	O=S(NC(N=N1)=CN1C2=CC=CC=C2)(C)=O
SM45	1,2,3-triazole	O=S(NC(N=N1)=CN1C2=CC=CC=C2)(C3=CC=CC=C3)=O
SM46	1,2,3-triazole	O=S(NC(N=N1)=CN1C2=CC=CC=C2)(N(C)C)=O

Compound Classes	Structures
acylsulfonamide	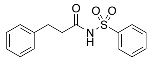 SM25 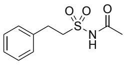 SM26
oxetane	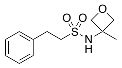 SM27 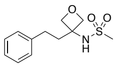 SM29 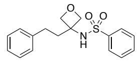 SM30 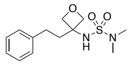 SM31
thietane	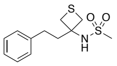 SM32 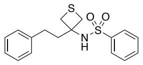 SM33 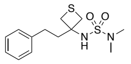 SM34
thietane-1-oxide	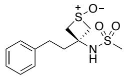 SM35 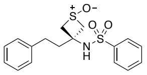 SM36 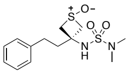 SM37
thietane-1,1-dioxide	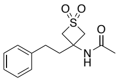 SM28 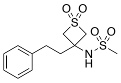 SM38 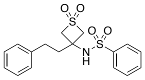 SM39 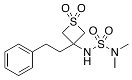 SM40
isoxazole	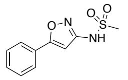 SM41 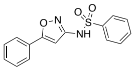 SM42 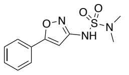 SM43
1,2,3-triazole	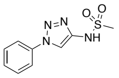 SM44 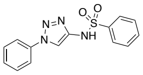 SM45 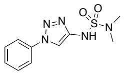 SM46

Figure S3. Compound classes and structures of the molecules in the SAMPL7 physical property challenge. SMILES of the compounds are in Table S3.

Table S7. Number of states per charge state for the microstates used in the SAMPL7 pK_a challenge. The total number of microstates (protomers and tautomers) is listed. Some of the molecules have up to 6 microstates, while others have only 2.

	**Charge State**				
	**+2**	**+1**	**0**	**-1**	**Total #**
SM25	0	1	3	2	6
SM26	0	1	3	2	6
SM27	0	0	1	1	2
SM28	0	1	2	2	5
SM29	0	0	1	1	2
SM30	0	0	1	1	2
SM31	0	1	1	1	3
SM32	0	0	1	1	2
SM33	0	0	1	1	2
SM34	0	1	1	1	3
SM35	0	0	2	3	5
SM36	0	0	2	3	5
SM37	0	2	2	2	6
SM38	0	0	1	1	2
SM39	0	0	1	1	2
SM40	0	1	1	1	3
SM41	0	1	1	1	3
SM42	0	1	2	1	4
SM43	1	2	2	1	6
SM44	0	1	1	1	3
SM45	0	1	1	1	3
SM46	1	2	1	1	5
